# Developing a new albuminuria-free risk prediction equation for kidney failure in patients with chronic kidney disease: retrospective cohort study

**DOI:** 10.1136/bmjmed-2025-001950

**Published:** 2026-02-02

**Authors:** Faye Cleary, David Prieto Merino, Rupert Major, Juan Jesus Carrero, Dorothea Nitsch

**Affiliations:** 1 Department of Non-Communicable Disease Epidemiology, London School of Hygiene and Tropical Medicine, London, UK; 2 Medical Research Council Integrative Epidemiology Unit, Population Health Sciences, University of Bristol, Bristol, UK; 3 Faculty of Medicine, University of Alcalá, Alcala de Henares, Spain; 4 Department of Cardiovascular Sciences, University of Leicester, Leicester, UK; 5 Department of Medical Epidemiology and Biostatistics, Karolinska Institutet, Stockholm, Sweden; 6 Division of Nephrology, Department of Clinical Sciences, Karolinska Institutet, Stockholm, Sweden

**Keywords:** Kidney failure, chronic, Epidemiology, Public health, Primary health care

## Abstract

**Objective:**

To develop new risk prediction equations for kidney failure in patients with chronic kidney disease who do not require data for the urine albumin to creatinine ratio.

**Design:**

Retrospective cohort study.

**Setting:**

Stockholm Creatinine Measurements (SCREAM) database of routinely collected electronic healthcare records in primary and outpatient care from the region of Stockholm, Sweden.

**Participants:**

116 158 adults with chronic kidney disease stages 3-4, defined by two estimated glomerular filtration rate (eGFR) results of <60 to ≥15 mL/min/1.73 m², at least 90 days apart, with no intermediate eGFR value ≥60 mL/min/1.73 m², between 1 January 2010 to 31 December 2018.

**Main outcome measure:**

Kidney failure, defined as starting kidney replacement therapy, recorded within five years of the index date.

**Results:**

Based on temporal split sample validation, development and validation cohorts included 85 012 patients (736 kidney replacement therapy events) and 28 338 patients (114 kidney replacement therapy events), respectively. After Cox regression with automated backwards selection, the final model included 10 predictors (in order of significance): eGFR, age, diabetes, sex, atrial fibrillation, antihypertensive drugs, peripheral artery disease, reduction in eGFR slope, acute kidney injury, and hypertension. Model discrimination was excellent in both the development cohort (C statistic 0.941, 95% confidence interval (CI) 0.932 to 0.951) and validation cohort (C statistic 0.944, 0.923 to 0.965). In 26 229 patients with data for the urine albumin to creatinine ratio, the four variable kidney failure risk equation (KFRE) showed marginal improvement in discrimination over our new equation (C statistic 0.950, 95% CI 0.942 to 0.958 for KFRE *v* 0.926, 0.915 to 0.936, for the new equation). KFRE under-estimated the risk in the study cohort, however, with an observed-expected event probability ratio of 2.11, suggesting that recalibration is required.

**Conclusions:**

The findings of the study indicate that predicting the risk of kidney failure with high accuracy in a general population of patients with chronic kidney disease is possible based on data that are routinely available, without requiring data for the urine albumin to creatinine ratio.

**WHAT IS ALREADY KNOWN ON THIS TOPIC**

- The four variable kidney failure risk equation (KFRE) was developed in 2011 with multinational validation in 2016, showing high discrimination for risk prediction of kidney failure in patients with chronic kidney disease
- KFRE is commonly used in routine care to identify patients at high risk of kidney failure who would benefit from increased monitoring, referral to nephrology care, or tailored interventions to reduce the risk of disease progression
- To predict the risk of kidney failure, KFRE requires data for urine albumin to creatinine ratio that are not routinely available for most patients with chronic kidney disease

**WHAT THIS STUDY ADDS**

- Only some patients with chronic kidney disease can be evaluated with KFRE because of the low routine testing rates for urine albumin to creatinine ratio
- A new equation is presented based on data that are likely to be routinely available in the general population of patients with chronic kidney disease
- The new equation and KFRE showed comparable discrimination performance in a large population cohort of chronic kidney disease in Stockholm, Sweden

**HOW THIS STUDY MIGHT AFFECT RESEARCH, PRACTICE, OR POLICY**

- After suitable external validation, the new risk prediction equation will allow risk prediction for kidney failure in the next five years, without the need for data for urine albumin to creatinine ratio
- Use of the new equation for risk stratification of patients with chronic kidney disease may improve equity of care

## Introduction

 Chronic kidney disease affects about 10% of the population worldwide (stages 1-5), and the number of people with chronic kidney disease is increasing.^1^,^6^ Later stage disease is associated with increased risks of cardiovascular events, mortality and, in rare instances, progression to end stage kidney disease requiring kidney replacement therapy, which places a substantial burden on healthcare services.^7^ Earlier identification of patients at highest risk and better targeted care efforts have the potential to delay disease progression and improve patient outcomes.

In the UK, most patients with chronic kidney disease are managed in primary care. The value of (often complex) prediction models (such as QRISK cardiovascular risk scores^8^) in primary care to aid decision making in the prioritisation of medical care has been increasingly recognised.^9^,^13^ In 2011, the kidney failure risk equation (KFRE) was developed to estimate the risk of kidney failure in people with pre-existing chronic kidney disease, requiring data on age, sex, estimated glomerular filtration rate (eGFR), and urine albumin to creatinine ratio.^14 15^ KFRE can be used to predict risks in the next two or five years. The four variable KFRE is currently recommended by the UK National Institute for Health and Care Excellence (NICE) to support risk assessment and decisions on referral to specialist care,^16^ with a risk threshold of 5% in the next five years indicating recommended referral.

In a 2016 audit in UK primary care, 54% of patients with diabetes and 30% with hypertension had a urine albumin to creatinine ratio (or protein to creatinine ratio) test in the past year or past five years, respectively.^17^ Similarly, a multinational meta-analysis from the Chronic Kidney Disease Prognosis Consortium (CKD-PC) found that 35% of patients with diabetes and 4% of patients with hypertension and no diabetes received urine albumin to creatinine ratio tests over a two year period.^18^ Lack of availability of urine albumin to creatinine ratio test results, which is not improving because of financial pressures on healthcare providers,^19^ prevents risk stratification with KFRE and may lead to barriers in further care for patients who are not tested. Other solutions are therefore needed to support general practitioners in identifying high risk patients and ensuring equality of access to healthcare services.

In this study, our aim was to develop new prognostic equations for kidney failure, suitable for use in all patients with chronic kidney disease stages 3-4, based on data that are routinely available (excluding results on urine albumin to creatinine ratio). The resulting equations may be used in clinical practice to support prioritisation of care and are particularly targeted to primary care settings, with the aim of improving prioritisation of further care.

## Methods

No protocol was prepared for this study and the study was not registered.

### Data sources

The Stockholm Creatinine Measurements (SCREAM) database was used to identify a retrospective cohort of patients with chronic kidney disease and for subsequent follow-up for outcomes. SCREAM has all kidney function tests conducted in routine care for all citizens in the region of Stockholm, Sweden.^20 21^ These data are linked to Swedish Renal Registry data for dates on starting kidney replacement therapy, and to other regional and national data sources for personal characteristics, clinical diagnoses recorded across all care settings, deaths, and dispensed prescription drugs.

The 2009 Chronic Kidney Disease Epidemiology Collaboration (CKD-EPI) formula (without adjustment for ethnic group) was used to estimate glomerular filtration rate in all analyses, and laboratories used standardised creatinine reporting throughout the study period. Only primary care and outpatient eGFR measures were used for identifying and analysing the cohort, because inpatient results may be affected by acute changes in kidney function which do not reflect long term chronic changes.

### Participants

Adults aged ≥18 years with chronic kidney disease stages 3-4, with two eGFR results ≥15 to <60 mL/min/1.73 m², at least 90 days apart, and with no intermediate eGFR ≥60 mL/min/1.73 m², were included in the study between 1 January 2010 and 31 December 2018. Although different cohort entry periods were considered (online supplemental table 1), we selected the most recent cohort (because of the high likelihood of the data reflecting current care practices and outcomes), with high predictor completeness (in particular, three historical test results for eGFR), reasonable availability of urine albumin to creatinine ratio (to allow comparisons with KFRE), and adequate kidney replacement therapy events (statistical power). The index date was defined as the date of the second qualifying eGFR result. Follow-up data were available from the index date to 31 December 2021. KFRE was developed and validated in a similar population of patients with chronic kidney disease stages 3-5, before starting any kidney replacement therapy.

### Outcome

The primary outcome was starting kidney replacement therapy, consistent with previous studies.^14 15^ A sensitivity outcome of non-rebounding eGFR <15 (aimed at detecting sustained chronic kidney disease stage 5) or kidney replacement therapy was defined as the first eGFR result <15 mL/min/1.73 m² that was not subsequently followed by an eGFR result ≥15 mL/min/1.73 m² at any later date (or starting kidney replacement therapy).

### Predictors

Candidate predictors were proposed based on clinical reasoning and were defined at the index date, unless otherwise specified. Predictors were: personal characteristics (age and sex); medical history (defined by ICD-10 (international classification of diseases, 10th revision) codes (online supplemental table 2) any time before the index date) of diabetes mellitus, hypertension, heart failure, coronary heart disease, atrial fibrillation, stroke, peripheral artery disease, or chronic obstructive pulmonary disease; renal history comprising baseline eGFR results, previous annual reduction in eGFR (estimated by simple linear regression models in individuals with at least three historical eGFR results up to and including the index date, where positive slopes indicate a reduction), and recent acute kidney injury event (hospital admission because of acute kidney injury (ICD-10) in the past year); and use of angiotensin converting enzyme inhibitors and angiotensin II receptor blockers (defined by Anatomical Therapeutic Chemical codes, prescribed at any time within the previous 12 months to three months after the index date).

Given recommendations to prescribe angiotensin converting enzyme inhibitors or angiotensin II receptor blockers in patients with chronic kidney disease with albuminuria,^16 22^ we anticipated that use of these drugs could be a proxy for albuminuria, in the absence of routine urine albumin to creatinine ratio testing. Continuous variables were included in models as linear covariates.

### Sample size

Sample size was based on feasibility and consideration of event numbers available for different cohort entry criteria (online supplemental table 1). In the selected cohort (development and validation combined), 870 kidney replacement therapy events were available over a five year period. Model development included 736 events (52 events for each candidate predictor).

### Missing data

The only missing data for the primary analyses was eGFR slope of decline, occurring in 2808 (2.4%) patients with fewer than three historical eGFR results. We performed a complete case analysis, and the small number of missing slope estimates likely did not affect the generalisability of the results. Follow-up for the sensitivity outcome (non-rebounding eGFR <15 or kidney replacement therapy) was affected by creatinine testing patterns. We evaluated testing patterns over time and by key risk factors to assess risks of ascertainment bias.

### Descriptive statistics

Study population characteristics were summarised according to the availability of data for urine albumin to creatinine ratio (within the previous 12 months to three months after the index date), to show subpopulations where risks could and could not be predicted with the four variable KFRE.

### Development versus validation

We used temporal split sample validation to divide the cohort in a 3:1 ratio into a development cohort (6 April 2010 to 7 January 2016) and validation cohort (7 January 2016 to 27 December 2018), respectively. This method preserved the bulk of the data for model fitting, while allowing ample data to precisely estimate model performance.

### Statistical analysis

We used Cox proportional hazards regression to develop a prognostic model for kidney failure, censored for death. This approach is the same as that used in the development of KFRE^14^ and means that model predicted risks do not account for the competing risk of death (ie, assuming that the risk of death is independent of the risk of kidney failure). Follow-up was capped at five years after the index date because of an intention to predict risks within the next five years (as for KFRE). We performed backward selection of predictors with automated variable selection (which was manually verified), removing variables with a P value <0.1 based on likelihood ratio tests. Preselection of candidate predictors based on clinical reasoning as well as the use of a large, representative, and comprehensive dataset reduced any risks of overfitting or selection of unimportant predictors by chance, justifying the use of backward selection as a suitable model building strategy with the goal of achieving a simple and clinically supported regression model. Reported hazard ratios also reflect adjusted associations observed in the data, helping clinical interpretability, whereas the LASSO (least absolute shrinkage and selection operator) model can sometimes result in shrinkage in estimates and therefore not necessarily represent true effects.

We used bootstrap resampling for internal validation in the development cohort with 200 bootstrap samples to evaluate model discrimination with the optimism corrected Harrell's C index (hence accounting for the possibility of over-optimistic apparent performance in the development dataset resulting from any model overfitting). Because of our large size of the dataset, average optimism (averaged differences between C statistics in the full sample and in the bootstrap samples) was close to zero, showing a robust modelling strategy that was not over-optimistic (not susceptible to overfitting), and a larger number of samples was not required to improve accuracy of estimation any further. We separately evaluated model discrimination in the validation cohort.

We used Stata command stcox for model development, with post-estimation with estat concordance, and confidence intervals computed from the somersd package.^23^ Methods for evaluation of model discrimination account for censoring for death or end of follow-up before event occurrence.

By incorporating evaluation of the baseline survival function at five years in the development cohort with the linear predictor function of the final Cox regression model, we provided an equation predicting the risk of kidney failure for an individual patient in the next five years. Estimated risks in the next five years assume that death will not occur during this time. We assessed calibration in the validation cohort by comparing Kaplan-Meier estimates of five year predicted risks (online supplemental information 1) (with predicted risk divided into five equal groups), with censoring for deaths.

Analysis code can be found at https://github.com/faye-cleary/SCREAM-risk-prediction. Analysis and reporting followed advice provided in Transparent Reporting of a Multivariable Prediction Model for Individual Prognosis or Diagnosis (TRIPOD) reporting guidelines^13^ (checklist available in online supplemental materials).

### Subgroup analyses

To evaluate model fairness, we assessed discrimination and calibration within subgroups, including by age, sex, diabetes status, chronic kidney disease stage, and availability of data on urine albumin to creatinine ratio. Subgroup analyses were carried out in the whole cohort, rather than the validation cohort, to increase precision of estimation because of interest in heterogeneity.

### Model comparisons

We compared our five year risk prediction equation with the four variable KFRE with recalibrated coefficients for non-North American populations^15^ (online supplemental information 2), assessing discrimination and calibration in patients with urine albumin to creatinine ratio data within the previous 12 months to three months after the index date. The nearest result for urine albumin to creatinine ratio to the index date was used. Histograms of linear predictions were produced by outcome status to visualise discrimination performance. We assessed correlation in linear predictions (and ranking of linear predictions) between the two models and distribution of differences in predicted risks. We produced Bland-Altman plots to visually assess agreement between the models. To assess the calibration of KFRE in our cohort, we computed the observed-expected event probability ratio, accounting for censoring.^12^

We also compared the performance of our final model as well as covariate hazard ratios with additional models containing age, sex, and eGFR coefficients only (the only readily available variables used in the KFRE) and with the full model without the acute kidney injury variable. The reasoning was to verify the additional clinical value of our model covariates beyond the basic KFRE variables (excluding urine albumin to creatinine ratio) and to weigh up the added clinical value of including acute kidney injury in the model against some concerns of data quality around this variable.

We had concerns about data quality in patients with previous acute kidney injury, both in the quality of capture of acute kidney injury in the dataset, which was based solely on ICD-10 codes which may be incomplete, and the fact that baseline eGFR values may not have represented steady state kidney function for patients recovering from a recent acute kidney injury. For example, a low (recovery) eGFR result at baseline may later have returned to a higher (steady state) eGFR value, meaning that patients with recent acute kidney injury may have been identified as having further progressed chronic kidney disease when in fact this was not the case. Another concern was that patients with a recent acute kidney injury would only be included in the analysis if they survived for long enough to subsequently have an outpatient eGFR result collected, representing a potentially healthier acute kidney injury subgroup (acute kidney injury survivors). This finding, however, likely reflects the reality of clinical care, where patients only present for risk assessment if they survive an acute kidney injury event.

### Clinical utility

We compared the clinical utility of our equation with KFRE with decision curve analysis and computed the difference in net benefit^24 25^ between the models for different risk thresholds of potential clinical interest.

### Patient and public involvement

This study involved secondary use of electronic healthcare records collected as part of routine clinical practice. The SCREAM database was designed for research purposes, with end goals to benefit patients through improvements in clinical practice. The current study did not involve direct engagement of patients or members of the public, but uses the laboratory tests and health trajectories of thousands of patients and we believe it is patient focused. We have liaised with patient representatives Miranda Scanlon (lay adviser group lead at Kidney Care UK) and Susan Lyon (chair of the UK Kidney Association Patient Council) on reporting of findings and plans for dissemination to patients.

## Results

### Participants

**Figure 1:**
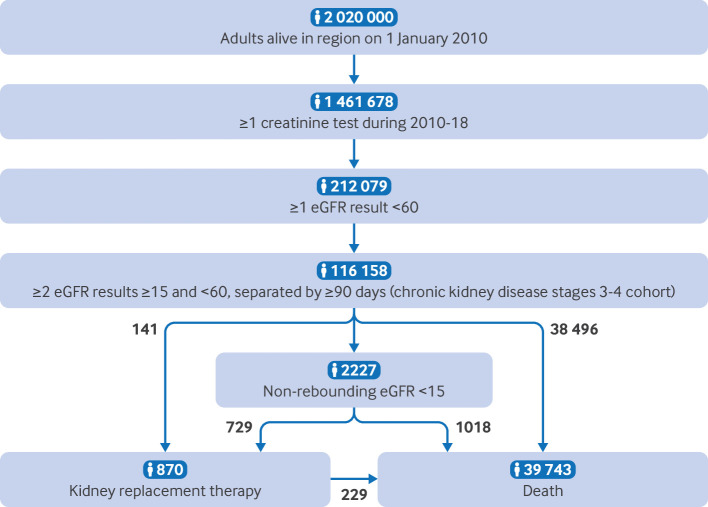
Flowchart of identifying the analysis cohort and subsequent outcome events in the next five years. Of those estimated to be living in the region at the beginning of the data collection period, about 72% had a creatinine test, 10% had at least one estimated glomerular filtration rate (eGFR) result <60 mL/min/1.73 m², and 6% met the selection criteria for chronic kidney disease stages 3-4 (excluding those who had already started kidney replacement therapy). Possible pathways for patient outcomes include: non-rebounding eGFR <15 only (n=480); non-rebounding eGFR <15 and kidney replacement therapy only (n=729); non-rebounding eGFR <15, kidney replacement therapy, and death (n=167); non-rebounding eGFR <15 and death only (n=1018); kidney replacement therapy only (n=79); kidney replacement therapy and death only (n=62); and death only (n=38 496). By adding combinations of this breakdown, total events for each outcome can be deduced, as shown in the flowchart

A total of 116 158 patients met the cohort entry criteria (figure 1 and online supplemental figure 1). Median time between qualifying eGFR tests was 7.0 months (online supplemental figure 2) and varied by chronic kidney disease stage (stage 3a=7.2 months, stage 3b=6.6 months, and stage 4=5.4 months). We identified 26 658 (23.0%) patients with a urine albumin to creatinine ratio test within the previous 12 months to three months after the index date. These patients were more likely to be men, of younger age, have later stage chronic kidney disease, diabetes, and hypertension, and be prescribed angiotensin converting enzyme inhibitors or ARB medications, representing a more morbid population (table 1).

**Table 1:** Baseline characteristics of patients with chronic kidney disease stages 3-4, overall and grouped by availability of data for urine albumin to creatinine ratio (within the previous 12 months to three months after the index date)

Characteristics	Chronic kidney disease stages 3-4		
	Total cohort (n=116 158)	No urine albumin to creatinine ratio data (n=89 500)	Urine albumin to creatinine ratio data (n=26 658)
Median (IQR) age (years)	79 (71-85)	80 (73-86)	74 (67-81)
Missing data	0	0	0
Women	66 429 (57.2)	54 158 (60.5)	12 271 (46.0)
Missing data	0	0	0
Diabetes	27 953 (24.1)	14 411 (16.1)	13 542 (50.8)
Hypertension	87 125 (75.0)	64 811 (72.4)	22 314 (83.7)
Coronary heart disease	12 287 (10.6)	9266 (10.4)	3021 (11.3)
Heart failure	27 418 (23.6)	27 418 (24.3)	5691 (21.4)
Atrial fibrillation	28 208 (24.3)	22 706 (25.4)	5502 (20.6)
Stroke	14 196 (12.2)	11 345 (12.7)	2851 (10.7)
Peripheral arterial disease	11 448 (9.9)	8546 (9.6)	2902 (10.9)
Chronic obstructive pulmonary disease	21 499 (18.5)	16 626 (18.6)	4873 (18.3)
Median (IQR) eGFR (mL/min/1.73 m²)	51 (44-56)	52 (44-56)	51 (42-56)
Chronic kidney disease stage:*			
3a	84 065 (72.4)	65 730 (73.4)	18 335 (68.8)
3b	25 873 (22.3)	19 859 (22.2)	6014 (22.6)
4	6220 (5.4)	3911 (4.4)	2309 (8.6)
eGFR frequency:†			
Median (IQR)	10 (7-16)	10 (6-15)	13 (8-20)
2 measures	2808 (2.4)	2378 (2.7)	430 (1.6)
3-5 measures	17 186 (14.8)	14 829 (16.6)	2357 (8.8)
≥6 measures	96 164 (82.8)	72 293 (80.7)	23 871 (89.6)
Median (IQR) eGFR coverage (years)	5.6 (4.2-8.2)	5.5 (4.2-7.9)	6.4 (4.3-9.3)
Median (IQR) previous reduction in eGFR slope (units/year)*	2.51 (1.20-4.27)	2.41 (1.12-4.11)	2.86 (1.50-4.77)
Missing (not computed)	2808 (2.4)	2361 (2.7)	447 (1.6)
Recent acute kidney injury	1795 (1.6)	875 (1.0)	920 (3.5)
Urine albumin to creatinine ratio (mg/g):			
Median (IQR)	NA	NA	1.7 (0.6-7.8)
<30	NA	NA	23 429 (87.9)
30-299	NA	NA	2866 (10.7)
≥300	NA	NA	362 (1.4)
Use of antihypertensive drugs	71 184 (61.3)	50 427 (56.3)	20 757 (77.9)

Values are number (%) unless indicated otherwise.

* Chronic kidney disease stage is based on estimated glomerular filtration rate (eGFR) value at the index date.

† Includes all measures between 2006 and 2018 before (and including) the index date, excluding inpatient measures.

eGFR, estimated glomerular filtration rate; IQR, interquartile range; NA, not available.

### Outcomes

A total of 96 427 (83.0%) patients had five years of follow-up data; the remaining 19 731 patients (17.0%) had at least three years of follow-up data, and median follow-up time was four years and two months (online supplemental figure 3). We identified 870 kidney replacement therapy events, 2227 non-rebounding eGFR <15 events, 2368 composite non-rebounding eGFR <15 or kidney replacement therapy events (sensitivity outcome), and 39 743 deaths during follow-up (figure 1 and online supplemental figure 4). Crude event rates were 1.9 events per 1000 patient years for kidney replacement therapy and 5.2 events per 1000 patient years for non-rebounding eGFR <15 or kidney replacement therapy. Event rates were higher in 2010 than in subsequent years because of identifying disproportionately more prevalent cases of chronic kidney disease (*v* incident cases) in the first year of follow-up (online supplemental figures 5-7), leading to higher outcome event rates in the development cohort than in the validation cohort.

#### Data completeness

The median number of eGFR records before kidney replacement therapy within the next five years after the index date was 7 (interquartile range 4-13). Before kidney replacement therapy, about 70-80% of patients with chronic kidney disease received eGFR tests in each calendar year, which varied by risk factors, and about 8% of patients died each year (online supplemental table 3).

#### Agreement between outcomes

Of 3723 patients with any eGFR result <15 mL/min/1.73 m², 759 (20.4%) started kidney replacement therapy compared with 729 of 2227 (32.7%) patients with non-rebounding eGFR <15 mL/min/1.73 m². Likewise, among the 870 patients starting kidney replacement therapy, 729 (84%) had previous non-rebounding eGFR <15 mL/min/1.73 m². Online supplemental figure 8 shows overlap of individual outcomes in the chronic kidney disease cohort. In the 729 patients with both events, median time between non-rebounding eGFR <15 and subsequent kidney replacement therapy was 9.3 months.

Patients with chronic kidney disease stage 5 who then started kidney replacement therapy mostly survived (25% died; median 8 days between eGFR <15 and death), whereas those not starting kidney replacement therapy mostly died (68% died; median 25 days between eGFR <15 and death). Reasons for not starting kidney replacement therapy are complex and likely contribute to the higher death rate in this group, such as an underlying greater risk of death, or perhaps because of older age or other significant risk factors, such as cancer.

### Model development

Of 116 158 identified patients, 2808 patients were excluded from the analysis because of missing results for eGFR slope of decline, leaving 850 kidney replacement therapy events and 2318 non-rebounding eGFR <15 or kidney replacement therapy events for analysis. The development and validation cohorts included 85 012 patients (736 kidney replacement therapy events) and 28 338 patients (114 kidney replacement therapy events), respectively (online supplemental table 4).

Predictors selected for the kidney replacement therapy prediction model (in order of significance) that increased the risk of kidney replacement therapy were: lower baseline eGFR value, younger age, history of diabetes, male sex, no history of atrial fibrillation, concurrent prescription of angiotensin converting enzyme inhibitors or angiotensin II receptor blockers, history of peripheral arterial disease, steeper reduction in eGFR, no recent acute kidney injury, and history of hypertension (table 2). We found similar results for the sensitivity outcome, but with some differences in variables included in the final models. Online supplemental information 1 shows the final prediction equations.

**Table 2:** Hazard ratios (95% confidence intervals) for new risk models for kidney replacement therapy (primary outcome) and non-rebounding estimated glomerular filtration rate (eGFR) <15 or kidney replacement therapy (sensitivity outcome) in the development cohort, with discrimination statistics evaluated in both the development and validation cohorts

Risk model	Hazard ratio (95% CI)	
	Kidney replacement therapy outcome	eGFR outcome
eGFR at baseline (per 5 mL/min/1.73 m^2^)	0.53 (0.51 to 0.54)	0.50 (0.49 to 0.51)
eGFR slope decline (per 5 mL/min/1.73 m^2^/year)	1.03 (1.01 to 1.06)	—
Acute kidney injury in past year	0.57 (0.36 to 0.89)	0.72 (0.56 to 0.91)
Age (per 10 year)	0.49 (0.47 to 0.51)	0.69 (0.67 to 0.71)
Female sex	0.54 (0.46 to 0.63)	0.56 (0.51 to 0.61)
Diabetes	2.15 (1.85 to 2.50)	1.45 (1.32 to 1.59)
Hypertension	1.26 (1.03 to 1.55)	—
Heart failure	—	1.14 (1.02 to 1.27)
Coronary heart disease	—	0.84 (0.73 to 0.97)
Atrial fibrillation	0.56 (0.43 to 0.74)	0.78 (0.69 to 0.89)
Peripheral arterial disease	1.50 (1.20 to 1.89)	1.26 (1.10 to 1.43)
Chronic obstructive pulmonary disease	—	—
Use of antihypertensive drugs	1.62 (1.29 to 2.04)	—
C statistic* (development cohort)	0.941 (0.932 to 0.951)	0.883 (0.875 to 0.892)
C statistic (validation cohort)	0.944 (0.923 to 0.965)	0.837 (0.808 to 0.866)

* Optimism corrected Harrell's C statistic, computed from bootstrap resampling.

CI, confidence interval.

### Model performance

**Figure 2:**
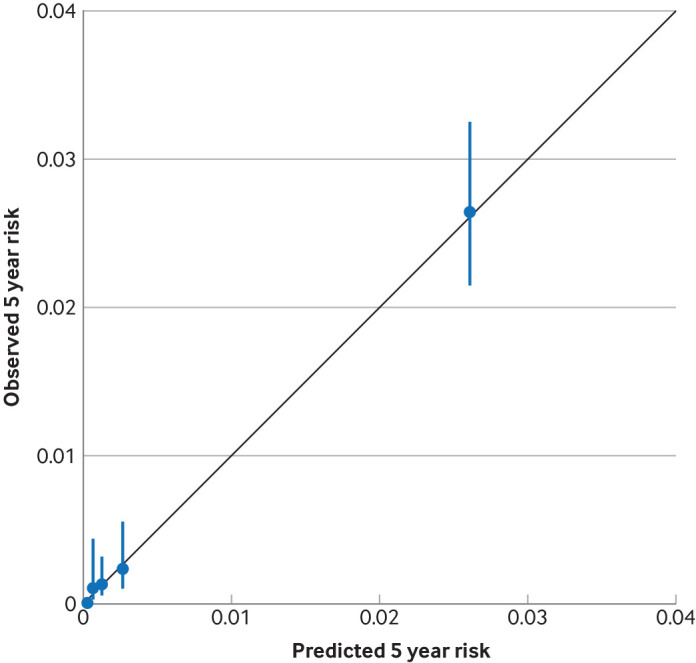
Observed versus predicted probability of kidney replacement therapy at five years, by predicted risk group (divided into five equal groups), in the validation cohort. Groups of predicted risk (presented as risks on a scale from 0 to 1) were 0-0.00049, 0.00049-0.00097, 0.00097-0.00188, 0.00188-0.00447, and 0.00447-1

In the primary (kidney replacement therapy) analysis, model discrimination was high in both the development and validation cohorts (table 2). Linear predictions showed good separation according to outcome status (online supplemental figure 9). Calibration plots showed no evidence of systematic under-estimation or over-estimation in the validation cohort (figure 2). The accuracy of the predicted risks seemed reasonably good within subgroups, with some small deviations from linearity (agreement between observed and expected risks; online supplemental figure 10). We found trends of under-estimation of risks in patients with urine albumin to creatinine ratio data and over-estimation in patients without urine albumin to creatinine ratio data, as well as over-estimation of risks in patients aged >80 years (although absolute differences between observed and predicted risks were small, with typically lower risks of kidney replacement therapy in those aged >80 years). Online supplemental table 5 shows the discrimination statistics for the new risk models by subgroup.

Model discrimination for prediction of the sensitivity outcome of non-rebounding eGFR <15 was good (table 2) but inferior to that observed in the primary analysis predicting starting kidney replacement therapy. Lower discrimination in the validation cohort may result from shorter follow-up duration for measurement of non-rebounding eGFR <15 or changes in eGFR testing patterns over time. We found a modest over-estimation of risks in the highest predicted risk group in the validation cohort (online supplemental figure 11).

### Comparison with four variable KFRE

Among 26 229 patients with data for urine albumin to creatinine ratio, KFRE under-estimated the risk of kidney replacement therapy on average, with an observed-expected event probability ratio of 2.11. Miscalibration seemed to be only in the highest predicted risk group (online supplemental figure 12). Despite this finding, discrimination was excellent (C statistic 0.950, 95% confidence interval 0.942 to 0.958), a marginal improvement on our new equation (C statistic 0.926, 0.915 to 0.936). Online supplemental figures 13 and 14 show linear prediction distributions.

The correlation between linear prediction scores comparing KFRE with our equation was 0.79 (online supplemental figure 15). Online supplemental figures 16 and 17 show the distribution of differences in predicted risks and per cent change in rank between equations. We found differences in the way these two equations predicted risk in individual patients with data for urine albumin to creatinine ratio (online supplemental table 6). Bland-Altman plots showed higher predicted risks with the new equation than with KFRE across the range of predicted risks, mainly because KFRE was miscalibrated and considerably under-estimated the risks in the population studied (online supplemental figure 18). Online supplemental figure 19 shows a small increase in net benefit (clinical utility) of using KFRE compared with our equation across the studied risk thresholds, in patients with data for urine albumin to creatinine ratio.

### Comparison with models with fewer covariates

The full model showed increased model performance compared with a three variable model (age, sex, and eGFR only), suggesting the important clinical value of additional model variables in predicting the risk of kidney failure (online supplemental table 7). Model performance was similar when we compared our full model with the model without acute kidney injury, and updated hazard ratios for other variables in the model were effectively unchanged, suggesting that removing acute kidney injury from the model would be reasonable on the basis of concerns about the quality of the data.

## Discussion

### Principal findings

Only 23% of patients with chronic kidney disease identified between 2010 and 2018 had a urine albumin to creatinine ratio result (required for risk prediction with KFRE) within the previous 12 months to three months after confirmed chronic kidney disease. Our equation predicted the risk of kidney replacement therapy with high discrimination and good calibration, without requiring data on urine albumin to creatinine ratio. In patients with data for urine albumin to creatinine ratio, the four variable KFRE showed marginal improvement in discrimination over our equation, but recalibration of KFRE is needed in the Swedish population of chronic kidney disease because of observed miscalibration.

### Strengths and weaknesses of this study

A major strength of this study was the SCREAM database used for analysis, which covered a large sample of the population in the Stockholm region with chronic kidney disease, including complete data for kidney function tests conducted in all care settings. The large sample size provided ample power to study the rare kidney replacement therapy outcome (which is clinically important, well defined, and reliably measured based on renal registry data), with precise estimation, and avoided any risk of overfitting. Only a few patients were excluded because of missing data, and the results are likely to be generalisable to Sweden and other white European populations with chronic kidney disease.

A weakness of this study was the lack of external validation in a different population or dataset. Trends of under-estimation in patients with data for urine albumin to creatinine ratio and over-estimation in patients without data for urine albumin to creatinine ratio also suggest that other factors not measured in our study are likely to exist which affect both clinical decisions to test for urine albumin to creatinine ratio and risk of kidney failure. Risk prediction may be further improved by including such factors (if routinely available). Risks were also over-estimated in those aged >80 years, although absolute differences between observed and predicted risks were small, with a low background risk of starting kidney replacement therapy in this group. This finding likely reflects decision making to more commonly decline kidney replacement therapy in very elderly people. Accuracy of estimation of historical eGFR slope of decline was limited by duration of coverage or number of tests in some patients, or both, meaning that we may fail to fully acquire information on the rate of reduction in eGFR. Incomplete eGFR data collection during follow-up (which varied over time and by risk factors) highlighted challenges in studying eGFR based outcomes for chronic kidney disease stage 5 with routinely collected data.

### Comparison with other studies

The original four variable KFRE was developed and validated in Canadian patients attending nephrology clinics during 2001-08,^14^ and was validated in a multinational meta-analysis of 31 cohorts participating in CKD-PC.^15^ In contrast, our analysis cohort represented a much broader population of patients with chronic kidney disease. Original KFRE validation had a C statistic of 0.91 (multinational validation: pooled C statistic 0.88), whereas our equation had a C statistic of 0.94 (but was limited to our original data source).

Compared with previous studies, our results are applicable to a broader population of patients with chronic kidney disease, not selected based on receiving tests for urine albumin to creatinine ratio tests or on referral to renal clinics. Also, our study used more recently collected data reflecting current care practices and population health. Our equation has not been validated in other cohorts with chronic kidney disease in different geographical locations, however, limiting widespread use. Where KFRE includes variables that are likely to be measured fairly consistently (age, sex, eGFR, and urine albumin to creatinine ratio), our equation includes variables which might be measured differently in different settings or depend on care practices that may change over time, affecting the transportability and longevity of our equation.

Similar to KFRE,^14 15^ we used Cox modelling censored for death for model development, which assumes non-informative censoring. This method assumes that conditional on covariate values, patients who die would have gone on to have had the same hazard of starting dialysis as those who do not die (had they not died). Other studies have used other modelling approaches^26^,^29^ that theoretically attempt to allow for the correlation of death and needing dialysis. The bias in risk estimation when assuming non-informative censoring is likely to be worse in those with a higher risk of death (eg, elderly people). Provided KFRE is correctly calibrated, in international cohort analyses, on average not much difference in predicted risks exists between models that take account of death and those that do not.^28^ When predicting risks in a general population of patients with chronic kidney disease, for example when identifying patients for referral to renal clinics, such small differences are likely to have little impact, given the low background risk of kidney failure.

### Study implications

By using data that are routinely available in the wider population with chronic kidney disease, and with demonstration of good model fairness between different patient subgroups, our equation has the potential to improve healthcare equity, by allowing risk prediction to be extended to patients not tested for urine albumin to creatinine ratio. Our risk prediction equation could be used in the Swedish population with chronic kidney disease (if adopted in clinical practice) to help treating physicians identify patients who may benefit from further care. This care might include prioritisation of urine albumin to creatinine ratio testing, drug prescribing, or referral to renal clinics. In the UK, a 5% risk threshold is used for identifying patients for referral.^16^

Individually reported hazard ratios for our model may not necessarily represent causal effects. For example, reduced hazards for those with atrial fibrillation and previous acute kidney injury may occur because of increased monitoring efforts, and only survivors of acute kidney injury were studied. Concerns about the quality of the data because of incomplete coding of acute kidney injury and measurement error in steady state (baseline) eGFR results among those recovering from a recent acute kidney injury may also explain the observed reduction in hazard associated with previous acute kidney injury, where a protective effect of acute kidney injury is not clinically plausible. Removing acute kidney injury from the final model would be reasonable on this basis before implementing in clinical practice, without incurring any meaningful effect on model performance. Increased hazards for those prescribed angiotensin converting enzyme inhibitors or angiotensin II receptor blockers likely occur because of increased prescribing in high risk patients. Although this finding may not be clinically intuitive, use of angiotensin converting enzyme inhibitors or angiotensin II receptor blockers as a proxy for other clinically important variables (such as urine albumin to creatinine ratio), which are missing in data sources, could be considered a strength of the model.

Consistency of model performance in both the development and validation cohorts suggests that healthcare provider behaviours involved in the model mechanism are likely to be entrenched over time, which is a sign of likely consistency of model performance in the future. Despite recommendations for urine albumin to creatinine ratio testing, uptake is only slowly improving,^17 30 31^ and therefore we cannot rely on urine albumin to creatinine ratio tests to help predict kidney failure. The use of other tools to help practitioners identify high risk patients is needed, especially because drugs are available to prevent the progression of chronic kidney disease, such as sodium-glucose cotransporter 2 inhibitors.

### Future research

We recommend that our model should be validated every few years on more recent data to ensure that predictions are still valid, with recalibration if required. External validation will be required in any other settings of intended use (assessing transportability).

### Conclusions

We have developed a powerful new tool for estimating the risk of kidney replacement therapy in the next five years for use in Swedish patients with chronic kidney disease stages 3-4, aimed at improving future risk stratification and prioritisation of care. Levels of albuminuria continue to be an important risk factor in patients with chronic kidney disease beyond its use in KFRE, and we strongly advocate for improved testing of urine albumin to creatinine ratio in routine care. Our equation offers an important opportunity to predict the risk of kidney replacement therapy in patients without recent results for urine albumin to creatinine ratio on record. In the absence of complete data on predictors included in our final model, the three variable KFRE is an alternative which shows good model performance in the population studied. Future work will assess transportability of the developed model to other settings.

## Supplementary material

10.1136/bmjmed-2025-001950 Supplementary file 1

10.1136/bmjmed-2025-001950 Supplementary file 2

## Data Availability

Data are available upon reasonable request.
