# Supplementary material for: Developing a new albuminuria-free risk prediction equation for kidney failure in patients with chronic kidney disease: retrospective cohort study
Source: BMJ Med. 2026 Feb 2;5(1):e001950. doi: 10.1136/bmjmed-2025-001950 (PMC12878267; doi:10.1136/bmjmed-2025-001950)
Supplement: Supplementary file 1 [file bmjmed-5-1-s001.pdf]

Supplementary Table 1: Frequency of identifiable CKD cases (2 x eGFR<60 over 90+ days) and availability of eGFR and albuminuria data at the time point of identification, over various time periods indicating possible analysis cohorts

	**2006-2014**	**2010-2014**	**2006-2018**	**2010-2018** (Final cohort)
Number of CKD patients identified in period	N = 104,048	N = 76,897	N = 140,991	N = 116,158
Number of eGFR tests up to and including date of confirmed CKD, median (IQR)	4 (2, 8)	9 (6, 14)	6 (3, 11)	10 (7, 16)
Number of patients with ≥ 3 eGFR tests as of index date	72,127 (69.3%)	75,238 (97.8%)	107,802 (76.5%)	113,350 (97.6%)
Number of patients with albuminuria testing within +/- 3 months of date of confirmed CKD				
Any albuminuria test[a]	**23,544 (22.6%)**	**17,340 (22.6%)**	**35,816 (25.4%)**	**30,304 (26.1%)**
uACR	10,164 (9.8%)	9,621 (12.5%)	18,245 (12.9%)	18,100 (15.6%)
PCR	1,514 (1.5%)	1,239 (1.6%)	2,247 (1.6%)	2,001 (1.7%)
Dipstick albumin	16,293 (15.7%)	11,217 (14.6%)	23,079 (16.4%)	18,395 (15.8%)
Number of patients with albuminuria testing within +/- 6 months of date of confirmed CKD				
Any albuminuria test[a]	**31,395 (30.2%)**	**23,276 (30.3%)**	**47,484 (33.7%)**	**40,296 (34.7%)**
uACR	14,036 (13.5%)	13,373 (17.4%)	24,706 (17.5%)	24,549 (21.1%)
PCR	2,353 (2.3%)	1,928 (2.5%)	3,515 (2.5%)	3,130 (2.7%)
Dipstick albumin	22,555 (21.7%)	15,627 (20.3%)	32,186 (22.8%)	25,851 (22.3%)
Number of patients with albuminuria testing within +/- 12 months of date of confirmed CKD				
Any albuminuria test[a]	**40,494 (38.9%)**	**30,465 (39.6%)**	**61,011 (43.3%)**	**52,173 (44.9%)**
uACR	19,037 (18.3%)	17,957 (23.4%)	32,739 (23.2%)	32,311 (27.8%)
PCR	3,394 (3.3%)	2,857(3.7%)	5,031 (3.6%)	4,552 (3.9%)
Dipstick albumin	30,013 (28.8%)	21,426 (27.9%)	43,455 (30.8%)	35,711 (30.7%)
Number of patients with albuminuria testing within **-12 months to +3 months** of date of confirmed CKD				
Any albuminuria test[a]	**33,530 (32.2%)**	**25,473 (33.1%)**	**51,071 (36.2%)**	**43,974 (37.9%)**
uACR	14,571 (14.0%)	14,538 (18.9%)	26,164 (18.6%)	26,658 (23.0%)
PCR	2,373 (2.3%)	2,037 (2.7%)	3,602 (2.6%)	3,307 (2.9%)
Dipstick albumin	24,137 (23.2%)	17,140 (22.3%)	34,833 (24.7%)	28,457 (24.5%)

Age[b]<br>18-39<br>40-59<br>60-79<br>80+	 539 (0.5%)<br>4,591 (4.4%)<br>46,506 (44.7%)<br>52,412 (50.4%)	 364 (0.5%)<br>3,099 (4.0%)<br>33,409 (43.5%)<br>40,025 (52.1%)	 762 (0.5%)<br>6,923 (4.9%)<br>67,356 (47.8%)<br>65,950 (46.8%)	 590 (0.5%)<br>5,487 (4.7%)<br>55,161 (47.5%)<br>54,920 (47.3%)
Female	61,233 (58.9%)	44,746 (58.2%)	81,343 (57.7%)	66,429 (57.2%)
CKD stage[b]<br>3a<br>3b<br>4	 73,095 (70.3%)<br>24,935 (24.0%)<br>6,018 (5.8%)	 51,133 (66.5%)<br>20,379 (26.5%)<br>5,385 (7.0%)	 104,263 (74.0%)<br>29,971 (21.3%)<br>6,757 (4.8%)	 84,065 (72.4%)<br>25,873 (22.3%)<br>6,220 (5.4%)
Diabetes[b]	23,447 (22.5%)	18,993 (24.7%)	31,944 (22.7%)	27,953 (24.1%)
Hypertension[b]	69,236 (66.5%)	57,865 (75.3%)	96,689 (68.6%)	87,125 (75.0%)
Total number of **KRT** events [and event rate]	1,972 (1.9%) [2.78 per 1000 py]	1,412 (1.8%) [2.98 per 1000 py]	2,170 (1.5%) [2.51 per 1000 py]	1,618 (1.4%) [2.53 per 1000 py]
Number of **KRT** events in next 5 years[c] [and event rate]	787 (0.8%) [1.90 per 1000 py]	695 (0.9%) [2.28 per 1000 py]	956 (0.7%) [1.71 per 1000 py]	870 (0.7%) [1.90 per 1000 py]
Total number of **eGFR < 15** events[d] [and event rate]	6,897 (6.6%) [9.90 per 1000 py]	4,985 (6.5%) [10.7 per 1000 py]	7,594 (5.4%) [8.91 per 1000 py]	5,729 (4.9%) [9.10 per 1000 py]
Number of **eGFR < 15** events in next 5 years[c,d] [and event rate]	3,781 (3.6%) [9.21 per 1000 py]	3,115 (4.1%) [10.3 per 1000 py]	4,415 (3.1%) [7.98 per 1000 py]	3,794 (3.3%) [8.38 per 1000 py]
Total number of **eGFR<15 events with no subsequent eGFR >= 15**[d] [and event rate]	4,600 (4.4%) [6.52 per 1000 py]	3,356 (4.4%) [7.13 per 1000 py]	5,020 (3.6%) [5.83 per 1000 py]	3,796 (3.3%) [5.97 per 1000 py]
Number of **eGFR<15 events in next 5 years, with no subsequent eGFR >= 15**[c,d] [and event rate]	2,159 (2.1%) [5.22 per 1000 py]	1,842 (2.4%) [6.06 per 1000 py]	2,524 (1.8%) [4.53 per 1000 py]	2,227 (1.9%) [4.89 per 1000 py]
Total number of **deaths** [and event rate]	72,644 (69.8%) [101 per 1000 py]	48,513 (63.1%) [101 per 1000 py]	83,161 (59.0%) [95.1 per 1000 py]	60,005 (51.7%) [93.1 per 1000 py]
Number of **deaths** in next 5 years[c] [and event rate]	38,835 (37.3%) [93.2 per 1000 py]	29,227 (38.0%) [95.4 per 1000 py]	48,479 (34.4%) [86.5 per 1000 py]	39,743 (34.2%) [86.6 per 1000 py]

[a]Number of patients with any test (uACR, PCR or dipstick). Note: This is not the sum of different types of albuminuria tests, as patients can have multiple different tests within the time window.
[b]Risk factors are defined at first biochemical evidence of CKD (2 x eGFR <60, separated by ≥90 days).
[c]Note: Outcomes censored at: 5 years since index date; end of follow up for outcomes (31st December 2021); or death, whichever occurs first. Note: Shorter follow up times will be available for cohort entry periods covering more recent years.
[d]Assessed using pre-KRT eGFR results only

Supplementary Table 2: ICD-10 codes (and ATC codes, where specified) for candidate predictor variables

**Comorbidity**	**ICD-10 codes (and additional ATC codes, where specified)**
Diabetes mellitus	E10-14
Hypertension	I10-15
Heart failure (HF)	I50
Coronary heart disease (CHD)	I21, I22
Atrial fibrillation (AF)	I48
Stroke (all-cause)	I60-64
Peripheral artery disease (PAD)	I70-73
Chronic obstructive pulmonary disorder (COPD)	I278, I279
Acute kidney injury (AKI)	N17
Use of ACEi/ARBs	ATC codes: C09A, C09B, C09C, C09D

Supplementary Table 3: Availability of creatinine data and follow up for outcomes in years following index date (excluding index date), in CKD analysis cohort, overall and stratified by factors which may predict likelihood of creatinine testing (leading to ascertainment bias)

	Year 1	Year 2	Year 3	Year 4	Year 5
**Number of patients starting follow up: N = 116,158**					
eGFR test recorded in year, N (%)	91,832 (79.1%)	87,581 (75.4%)	79,488 (68.4%)	70,930 (61.1%)	58,271 (50.2%)
Pre-KRT eGFR test recorded in year, N (%)	91,829 (79.1%)	87,498 (75.3%)	79,295 (68.3%)	70,617 (60.8%)	57,840 (49.8%)
**Number at risk at beginning of year (following index date)** [a]	**N = 116,158**	**N = 106,482**	**N = 97,809**	**N = 89,840**	**N = 82,347**
eGFR test recorded in year if at risk at beginning of year, N (%)	91,832 (79.1%)	87,509 (82.2%)	79,305 (81.1%)	70,630 (78.6%)	57,852 (70.3%)
Pre-KRT eGFR test recorded in year if at risk at beginning of year, N (%)	91,829 (79.1%)	87,498 (82.2%)	79,295 (81.1%)	70,615 (78.6%)	57,840 (70.2%)
eGFR test frequency per year if at risk at beginning of year, Median (IQR)[b]	2 (1, 3)	2 (1, 3)	2 (1, 3)	2 (1, 3)	1 (0, 3)
KRT event in year, N (%)	96 (0.08%)	143 (0.13%)	177 (0.18%)	214 (0.24%)	240 (0.29%)
Non-rebounding eGFR<15 event in year, N (%)	409 (0.35%)	504 (0.47%)	456 (0.47%)	442 (0.49%)	416 (0.51%)
Deaths in year, N (%)	9,600 (8.3%)	8,551 (8.0%)	7,845 (8.0%)	7,342 (8.2%)	6,405 (7.8%)
**Diabetes:**<br>Number at risk	**N = 27,953**	**N = 25,557**	**N = 23,272**	**N = 21,215**	**N = 19,179**
Pre-KRT eGFR test recorded	24,004 (85.9%)	22,732 (88.9%)	20,396 (87.6%)	18,203 (85.8%)	14,609 (76.2%)
KRT event	48 (0.17%)	77 (0.30%)	83 (0.36%)	93 (0.44%)	103 (0.54%)

**uACR tested:**<br>Number at risk	**N = 26,658**	**N = 25,206**	**N = 23,698**	**N = 22,200**	**N = 20,669**
Pre-KRT eGFR test recorded	23,247 (87.2%)	22,594 (89.6%)	20,872 (88.1%)	18,758 (84.5%)	14,674 (71.0%)
KRT event	77 (0.29%)	115 (0.46%)	140 (0.59%)	155 (0.70%)	179 (0.87%)
**uACR not tested:**<br>Number at risk	**N = 89,500**	**N = 81,276**	**N = 74,111**	**N = 67,640**	**N = 61,678**
Pre-KRT eGFR test recorded	68,582 (76.6%)	64,904 (79.9%)	58,423 (78.8%)	51,857 (76.7%)	43,166 (70.0%)
KRT event	19 (0.02%)	28 (0.03%)	37 (0.05%)	59 (0.09%)	61 (0.10%)
**CKD stage 3a:**<br>Number at risk	**N = 84,065**	**N = 79,094**	**N = 73,980**	**N = 69,111**	**N = 64,408**
Pre-KRT eGFR test recorded	65,113 (77.5%)	64,828 (82.0%)	59,870 (80.9%)	53,936 (78.9%)	44,224 (68.7%)
KRT event	14 (0.02%)	15 (0.02%)	29 (0.04%)	41 (0.06%)	65 (0.10%)
**CKD stage 3b:**<br>Number at risk	**N = 25,873**	**N = 22,507**	**N = 19,836**	**N = 17,495**	**N = 15,326**
Pre-KRT eGFR test recorded	21,331 (82.4%)	18,502 (82.2%)	16,060 (81.0%)	14,005 (80.1%)	11,547 (75.3%)
KRT event	15 (0.06%)	30 (0.13%)	58 (0.29%)	77 (0.44%)	74 (0.48%)
**CKD stage 4:**<br>Number at risk	**N = 6,220**	**N = 4,881**	**N = 3,993**	**N = 3,234**	**N = 2,613**
Pre-KRT eGFR test recorded	5,385 (86.6%)	4,168 (85.4%)	3,365 (84.3%)	2,674 (82.7%)	2,069 (79.2%)
KRT event	67 (1.08%)	98 (2.01%)	90 (2.25%)	96 (2.97%)	101 (3.87%)

[a]To be at risk at beginning of year, patient must be alive and not have started KRT
[b]Results shown for all patients, including those with no eGFR tests in year

Supplementary Table 4: Patient characteristics in development and validation cohorts

Patient characteristics	Development cohort	Validation cohort
N	**N = 85,012**	**N = 28,338**
Age, median (IQR)	80 (72, 86)	76 (70, 83)
Sex female, n (%)	49,158 (57.8%)	15,548 (54.9%)
Diabetes, n (%)	21,117 (24.8%)	6,587 (23.2%)
Hypertension, n (%)	64,316 (75.7%)	21,378 (75.4%)
CHD, n (%)	9,596 (11.3%)	2,572 (9.1%)
HF, n (%)	21,790 (25.6%)	5,329 (18.8%)
AF, n (%)	21,072 (24.8%)	6,750 (23.8%)
Stroke, n (%)	10,924 (12.9%)	3,055 (10.8%)
PAD, n (%)	8,875 (10.4%)	2,469 (8.7%)
COPD, n (%)	15,567 (18.3%)	5,662 (20.0%)
eGFR, median (IQR)	50 (42, 55)	54 (49, 57)
CKD stage, n (%)		
3a	58,155 (68.4%)	24,054 (84.9%)
3b	21,353 (25.1%)	3,727 (13.2%)
4	5,504 (6.5%)	557 (2.0%)
Prior decline in eGFR slope (units per year)[a]	2.62 (1.09, 4.61)	2.33 (1.42, 3.46)
Recent AKI	1,134 (1.3%)	624 (2.2%)
Use of ACEi/ARBs	51,857 (61.0%)	18,062 (63.7%)
KRT, N (rate)	736 (2.17 per 1000 py)	114 (1.07 per 1000 py)
Deaths, N (rate)	31,536 (92.5 per 1000 py)	7,289 (68.5 per 1000 py)

[a]Includes all measures between 2006-2018 prior to (and including) the index date, excluding inpatient measures

Supplementary Table 5: Discrimination statistics for new risk models, by sup-population, evaluated in all patients (development and validation cohorts combined).

**Patient population**	**C statistic (95% CI)[a]**	
	**KRT model**	**eGFR outcome model**
Entire CKD cohort	0.942 (0.933, 0.951)	0.880 (0.871, 0.888)
Sex		
Male	0.929 (0.917, 0.941)	0.877 (0.866, 0.888)
Female	0.945 (0.929, 0.961)	0.869 (0.855, 0.884)
Age, years		
<80	0.932 (0.0.923, 0.941)	0.903 (0.894, 0.911)
≥80	0.837 (0.750, 0.925)	0.840 (0.823, 0.857)
CKD stage		
3a	0.855 (0.821, 0.889)	0.691 (0.664, 0.719)
3b	0.898 (0.879, 0.917)	0.737 (0.716, 0.757)
4	0.865 (0.850, 0.880)	0.762 (0.748, 0.776)
Diabetes		
Yes	0.938 (0.924, 0.952)	0.873 (0.861, 0.884)
No	0.930 (0.918, 0.942)	0.875 (0.863, 0.888)
Albuminuria data		
Yes	0.926 (0.915, 0.936)	0.893 (0.883, 0.902)
No	0.897 (0.872, 0.922)	0.836 (0.821, 0.851)
3x2 breakdown by age and CKD stage		
CKD stage / Age[b]		
3a, <80	0.842 (0.807, 0.878)	0.767 (0.740, 0.795)
3b, <80	0.833 (0.805, 0.860)	0.776 (0.755, 0.797)
4, <80	0.772 (0.749, 0.794)	0.737 (0.719, 0.755)
3a, ≥80	0.631 (0.465, 0.797)	0.574 (0.527, 0.622)
3b, ≥80	0.948 (0.918, 0.977)	0.648 (0.611, 0.686)
4, ≥80	0.841 (0.759, 0.924)	0.760 (0.736, 0.785)

[a]Discrimination statistics by subgroup should be interpreted with caution. Lower C statistics are expected within subgroups due to less variation (less heterogeneity in predictor values) which makes it more difficult for the model to discriminate between outcomes. Calibration plots are more useful for subgroup comparisons of model performance.

[b]Sample size for CKD stage / age strata for computation of discrimination statistics were as follows:
3a, <80: N = 46,980 (41.5%); 3b, <80: N = 10,426 (9.2%); 4, <80: N = 2,401 (2.1%);
3a, ≥80: N = 35,229 (31.1%); 3b, ≥80: N = 14,654 (12.9%); 4, ≥80: N = 3,660 (3.2%).

Supplementary Table 6: Patient characteristics by (A) quintile of model predicted risk and (B) whether predicted risk >5%, separately for new equation and KFRE, in the subset of patients with uACR data within -12 months to +3 months of the index date

(A)

	Q1:<br>New equation (P, 0 – 0.001)<br>KFRE (P not shown; miscalibrated)		Q2:<br>New equation (P, 0.001 – 0.003)<br>KFRE (P not shown; miscalibrated)		Q3:<br>New equation (P, 0.003 – 0.006)<br>KFRE (P not shown; miscalibrated)		Q4:<br>New equation (P, 0.006 – 0.018)<br>KFRE (P not shown; miscalibrated)		Q5:<br>New equation (P, 0.018 - 1)<br>KFRE (P not shown; miscalibrated)	
New equation	Age, median (IQR)	81 (76,86)	Age, median (IQR)	76 (71, 81)	Age, median (IQR)	73 (68, 79)	Age, median (IQR)	71 (64, 77)	Age, median (IQR)	67 (57, 76)
	Female, N (%)	3,649 (69.6%)	Female, N (%)	2,717 (51.8%)	Female, N (%)	2,131 (40.6%)	Female, N (%)	1,912 (36.5%)	Female, N (%)	1,656 (31.6%)
	Diabetes, N (%)	1,400 (26.7%)	Diabetes, N (%)	2,581 (59.2%)	Diabetes, N (%)	3,164 (60.3%)	Diabetes, N (%)	3,285 (62.6%)	Diabetes, N (%)	2,993 (57.1%)
	Hyp, N (%)	4,111 (78.4%)	Hyp, N (%)	4,418 (84.2%)	Hyp, N (%)	4,560 (86.9%)	Hyp, N (%)	4,501 (86.8%)	Hyp, N (%)	4,452 (84.9%)
	CKD stage, N (%)		CKD stage, N (%)		CKD stage, N (%)		CKD stage, N (%)		CKD stage, N (%)	
	3a	5,158 (89.3%)	3a	4,861 (92.7%)	3a	4,343 (82.8%)	3a	3,012 (57.4%)	3a	713 (13.6%)
	3b	88 (1.7%)	3b	382 (7.3%)	3b	887 (16.9%)	3b	2,062 (39.3%)	3b	2,467 (47.0%)
	4	0	4	4 (0.1%)	4	16 (0.3%)	4	172 (3.3%)	4	2,065 (39.3%)
	uACR (mg/g)		uACR (mg/g)		uACR (mg/g)		uACR (mg/g)		uACR (mg/g)	
	median (IQR)	0.90 (0.50, 2.40)	median (IQR)	0.90 (0.45, 2.90)	median (IQR)	1.20 (0.50, 18.3)	median (IQR)	1.80 (0.60, 7.90)	median (IQR)	6.90 (1.38, 39.5)
	<30	5,130 (97.8%)	<30	5,029 (95.9%)	<30	4,887 (93.2%)	<30	4,649 (88.6%)	<30	3,754 (71.6%)
	30-299	108 (2.1%)	30-299	203 (3.9%)	30-299	342 (6.5%)	30-299	541 (10.3%)	30-299	1,317 (25.1%)
	≥300	8 (0.2%)	≥300	14 (0.3%)	≥300	17 (0.3%)	≥300	56 (1.1%)	≥300	174 (3.3%)
KFRE	Age, median (IQR)	76 (71, 81)	Age, median (IQR)	75 (69, 80)	Age, median (IQR)	75 (68, 81)	Age, median (IQR)	74 (66, 80)	Age, median (IQR)	70 (60, 79)
	Female, N (%)	3,323 (63.3%)	Female, N (%)	2,645 (50.4%)	Female, N (%)	2,244 (42.8%)	Female, N (%)	2,047 (39.0%)	Female, N (%)	1,806 (34.4%)
	Diabetes, N (%)	2,481 (47.3%)	Diabetes, N (%)	2,684 (51.2%)	Diabetes, N (%)	2,861 (54.5%)	Diabetes, N (%)	2,877 (54.8%)	Diabetes, N (%)	2,520 (48.1%)
	Hyp, N (%)	4,439 (84.6%)	Hyp, N (%)	4,418 (84.2%)	Hyp, N (%)	4,450 (84.8%)	Hyp, N (%)	4,452 (84.9%)	Hyp, N (%)	4,283 (81.7%)
	CKD stage, N (%)		CKD stage, N (%)		CKD stage, N (%)		CKD stage, N (%)		CKD stage, N (%)	
	3a	5,235 (99.8%)	3a	5,200 (99.1%)	3a	4,350 (82.9%)	3a	2,617 (49.9%)	3a	685 (13.1%)
	3b	11 (0.2%)	3b	46 (0.9%)	3b	895 (17.1%)	3b	2,567 (48.9%)	3b	2,367 (45.1%)
	4	0	4	0	4	1 (0.02%)	4	62 (1.2%)	4	2,193 (41.8%)
	uACR (mg/g)		uACR (mg/g)		uACR (mg/g)		uACR (mg/g)		uACR (mg/g)	
	median (IQR)	0.45 (0.30, 0.70)	median (IQR)	0.80 (0.48, 1.55)	median (IQR)	1.70 (0.70, 4.00)	median (IQR)	3.90 (1.30, 12.7)	median (IQR)	18.1 (4.60, 73.0)
	<30	5,246 (100%)	<30	5,246 (100%)	<30	5,215 (99.4%)	<30	4,631 (88.3%)	<30	3,111 (59.3%)
	30-299	0	30-299	0	30-299	31 (0.6%)	30-299	607 (11.6%)	30-299	1,873 (35.7%)
	≥300	0	≥300	0	≥300	0	≥300	8 (0.2%)	≥300	261 (5.0%)

(B)

	P < 5% (based on individual equation predicted risk)		P >= 5% (based on individual equation predicted risk)	
New equation		[N = 23,709]		[N = 2,520]
	Age, median (IQR)	75 (68, 81)	Age, median (IQR)	64 (52, 73)
	Female, N (%)	11,331 (47.8%)	Female, N (%)	734 (29.1%)
	Diabetes, N (%)	12,045 (50.8%)	Diabetes, N (%)	1,378 (54.7%)
	Hypertension, N (%)	19,923 (84.0%)	Hypertension, N (%)	2,119 (84.1%)
	CKD stage, N (%)		CKD stage, N (%)	
	3a	17,978 (75.8%)	3a	109 (4.3%)
	3b	5,000 (21.1%)	3b	886 (35.2%)
	4	731 (3.1%)	4	1,525 (60.5%)
	uACR (mg/g)		uACR (mg/g)	
	median (IQR)	1.20 (0.50, 4.70)	median (IQR)	13.8 (2.50, 70.8)
	<30	21,895 (92.4%)	<30	1,554 (61.7%)
	30-299	1,669 (7.0%)	30-299	842 (33.4%)
	≥300	145 (0.6%)	≥300	124 (4.9%)
KFRE		[N = 24,511]		[N = 1,718]
	Age, median (IQR)	74 (68, 81)	Age, median (IQR)	67 (55, 77)
	Female, N (%)	11,548 (47.1%)	Female, N (%)	517 (30.1%)
	Diabetes, N (%)	12,676 (51.7%)	Diabetes, N (%)	747 (43.5%)
	Hypertension, N (%)	20,629 (84.2%)	Hypertension, N (%)	1,413 (82.3%)
	CKD stage, N (%)		CKD stage, N (%)	
	3a	18,070 (73.7%)	3a	17 (1.0%)
	3b	5,452 (22.2%)	3b	434 (25.3%)
	4	989 (4.1%)	4	1,267 (73.8%)
	uACR (mg/g)		uACR (mg/g)	
	median (IQR)	1.20 (0.50, 4.50)	median (IQR)	57.1 (17.5, 151.9)
	<30	22,847 (93.2%)	<30	602 (35.1%)
	30-299	1,568 (6.4%)	30-299	943 (54.9%)
	≥300	96 (0.39%)	≥300	173 (10.1%)

It is reassuring that there are fairly similar distributions of patient characteristics among the “high” risk, when categorised by our new equation compared to when categorised by KFRE. This suggests that, while our equation does NOT utilise uACR data, it appears able to proxy for it reasonably well using other variables. When we recall that KFRE in fact under-estimates risk in our population, we expect to therefore see more higher risk patients in the “>5% risk” group for KFRE than for the new equation which is well calibrated. This may partially explain why we observe lower eGFR and higher uACR in the KFRE high risk group compared to the new equation high risk group.

Supplementary Table 7: Comparison of risk prediction models for KRT, demonstrating the clinical value of inclusion of covariates beyond the 3-variable KFRE coefficients (age, sex, eGFR) and the clinical value of AKI (which could be removed for data quality reasons)

	Hazard ratios for KRT (95% CIs)		
	**3-variable KFRE coefficients (age, sex, eGFR)**	**Full model**	**Full model without AKI**
eGFR at baseline, per 5 mL/min/1.73m^2	0.52 (0.50 to 0.54)	0.53 (0.51 to 0.54)	0.53 (0.51 to 0.54)
eGFR slope decline, per 5 mL/min/1.73m^2/yr	-	1.03 (1.01 to 1.06)	1.03 (1.01 to 1.05)
Acute kidney injury in last year	-	0.57 (0.36 to 0.89)	-
Age, per 10 yr	0.50 (0.48 to 0.52)	0.49 (0.47 to 0.51)	0.49 (0.47 to 0.64)
Female sex	0.52 (0.45 to 0.61)	0.54 (0.46 to 0.63)	0.54 (0.46 to 0.64)
Diabetes	-	2.15 (1.85 to 2.50)	2.15 (1.85 to 2.51)
Hypertension	-	1.26 (1.03 to 1.55)	1.28 (1.04 to 1.57)
Atrial fibrillation	-	0.56 (0.43 to 0.74)	0.56 (0.43 to 0.73)
Peripheral Arterial disease	-	1.50 (1.20 to 1.89)	1.51 (1.20 to 1.90)
Use of anti-hypertensives	-	1.62 (1.29 to 2.04)	1.63 (1.29 to 2.05)
C statistic (development cohort)	0.936 (0.926 to 0.945)	0.941 (0.932 to 0.951)	0.941 (0.932 to 0.951)
C statistic (validation cohort)	0.936 (0.913 to 0.959)	0.944 (0.923 to 0.965)	0.944 (0.924 to 0.965)

Supplementary Figure 1. Flow chart of analysis cohort identification and number of outcome events, with event numbers shown in the analysis population after removal of those with missing eGFR slope data. Outcome events and deaths include those recorded within the next 5 years post index date. Breakdown of deaths after KRT in those with complete eGFR slope data: 165 had eGFR < 15 outcome; 61 did not have eGFR < 15 outcome.

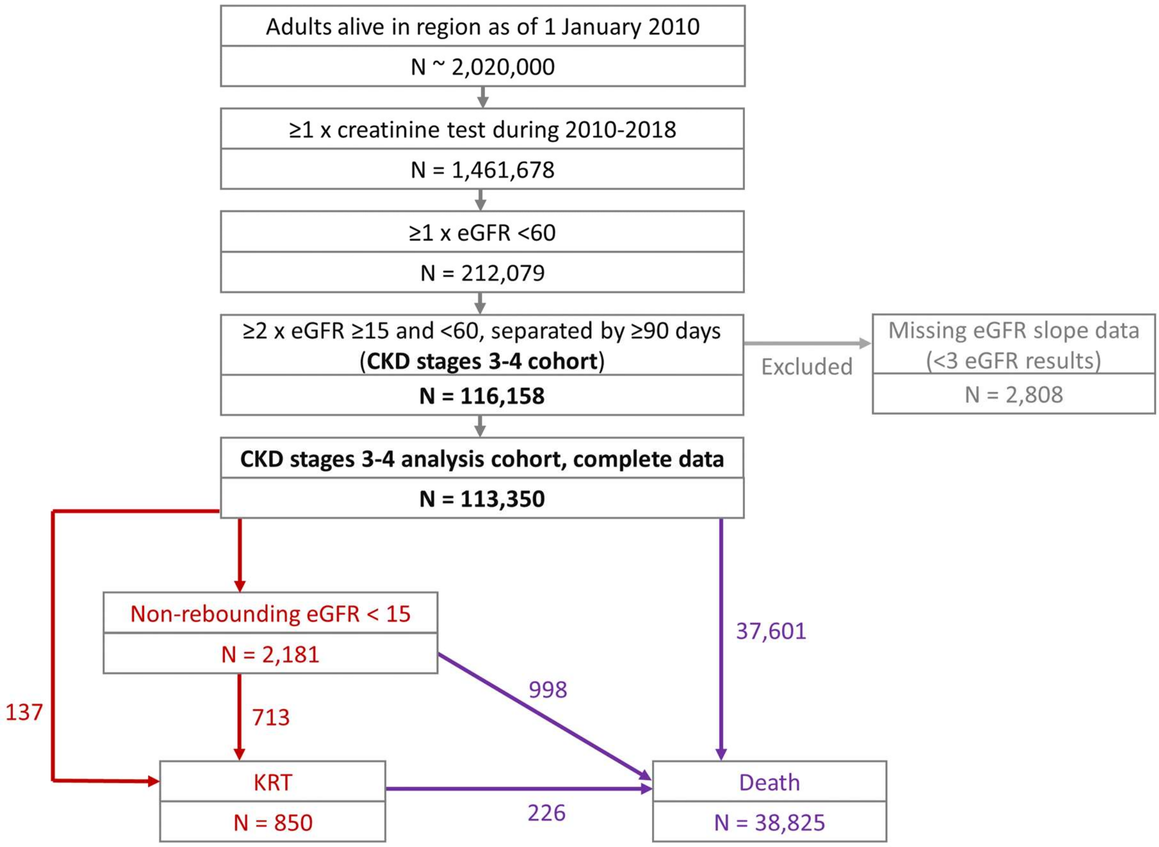

Supplementary Figure 2: Distribution of (A) time between qualifying eGFR results and (B) year of index date, in entire CKD cohort

(A)

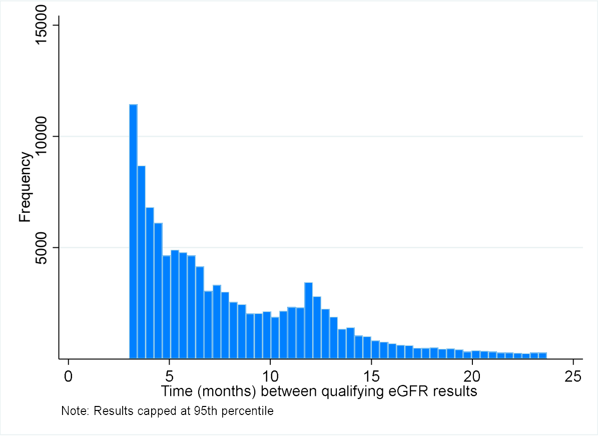

(B)

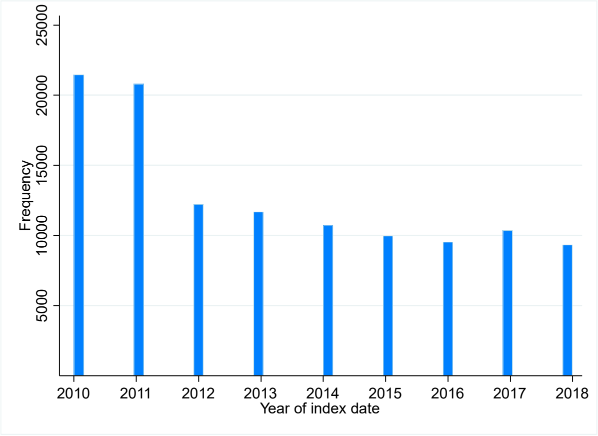

Supplementary Figure 3: Available follow-up time (years) between index date and end of outcomes data collection capped at 5 years in entire CKD cohort, by analysis cohort (development, validation)

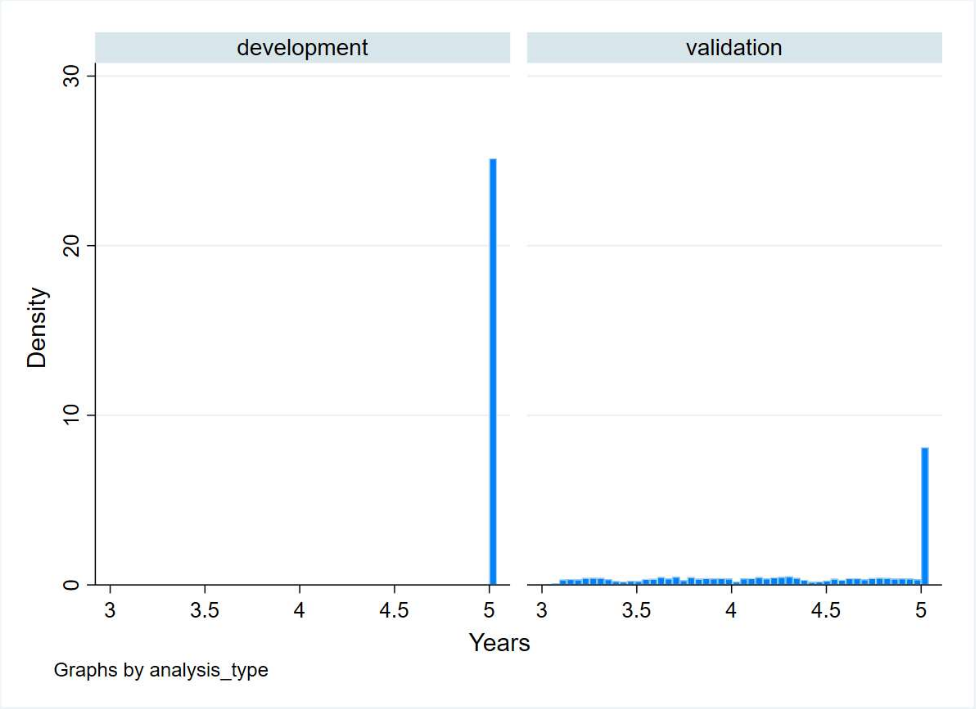

Supplementary Figure 4: Histograms of outcome events (and censoring variables) by year: (A) KRT; (B) non-rebounding eGFR<15; (C) Pre-KRT deaths. Analysis includes only those events up to 5 years post index date.

(A)

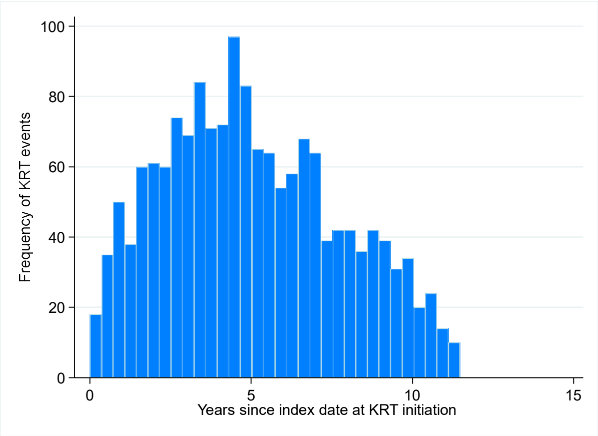

(B)

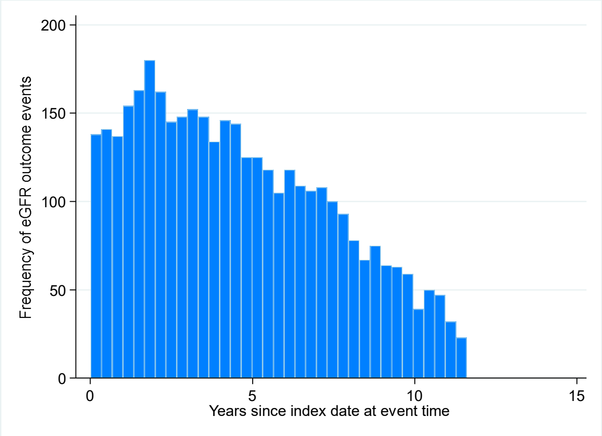

(C)

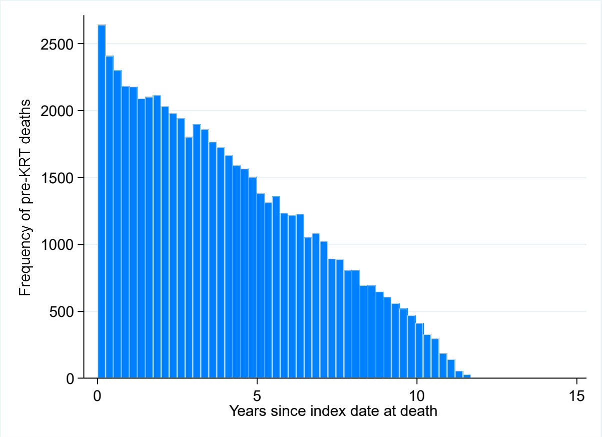

Supplementary Figure 5: Kaplan Meier failure curves for (A) main outcome (KRT) and (B) sensitivity outcome (non-rebounding eGFR < 15 or KRT), in entire CKD cohort

(A)

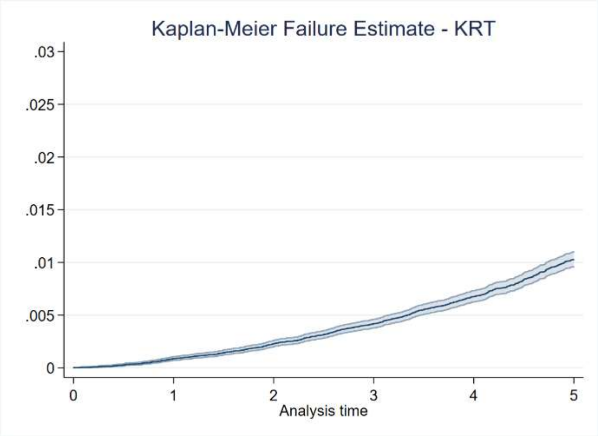

(B)

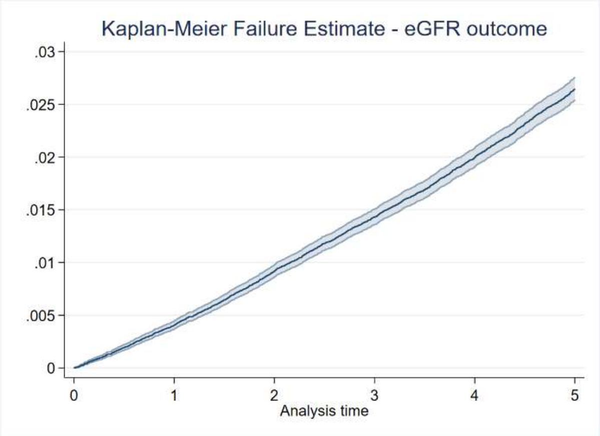

Supplementary Figure 6: Kaplan Meier failure curves for (A) main outcome (KRT) and (B) sensitivity outcome (non-rebounding eGFR < 15 or KRT), by analysis cohort (development, validation)

(A) **(B)**

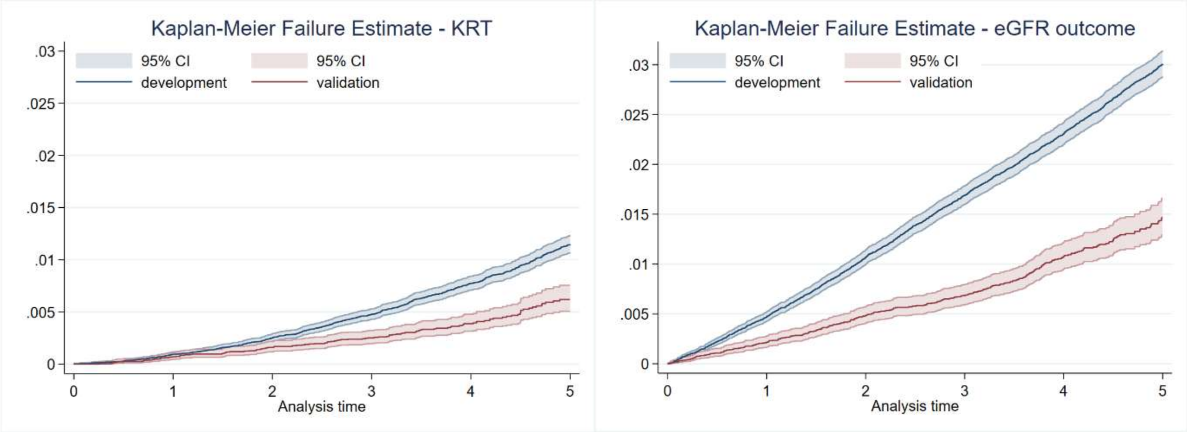

Supplementary Figure 7: Kaplan Meier failure curves for (A) main outcome (KRT) and (B) sensitivity outcome (non-rebounding eGFR < 15 or KRT), by year of index date

(A)

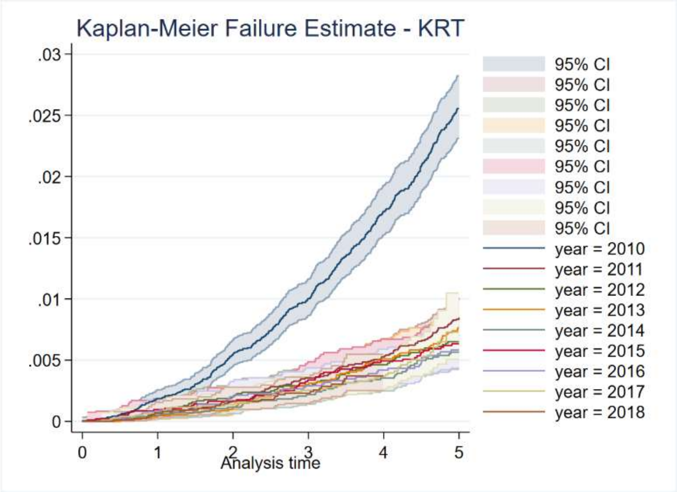

(B)

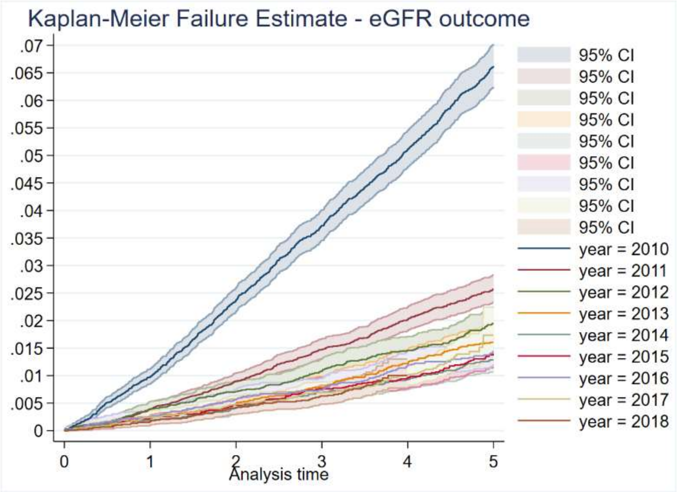

Supplementary Figure 8: To-scale venn diagram displaying overlap in outcomes experienced by the CKD cohort in a 5 year period

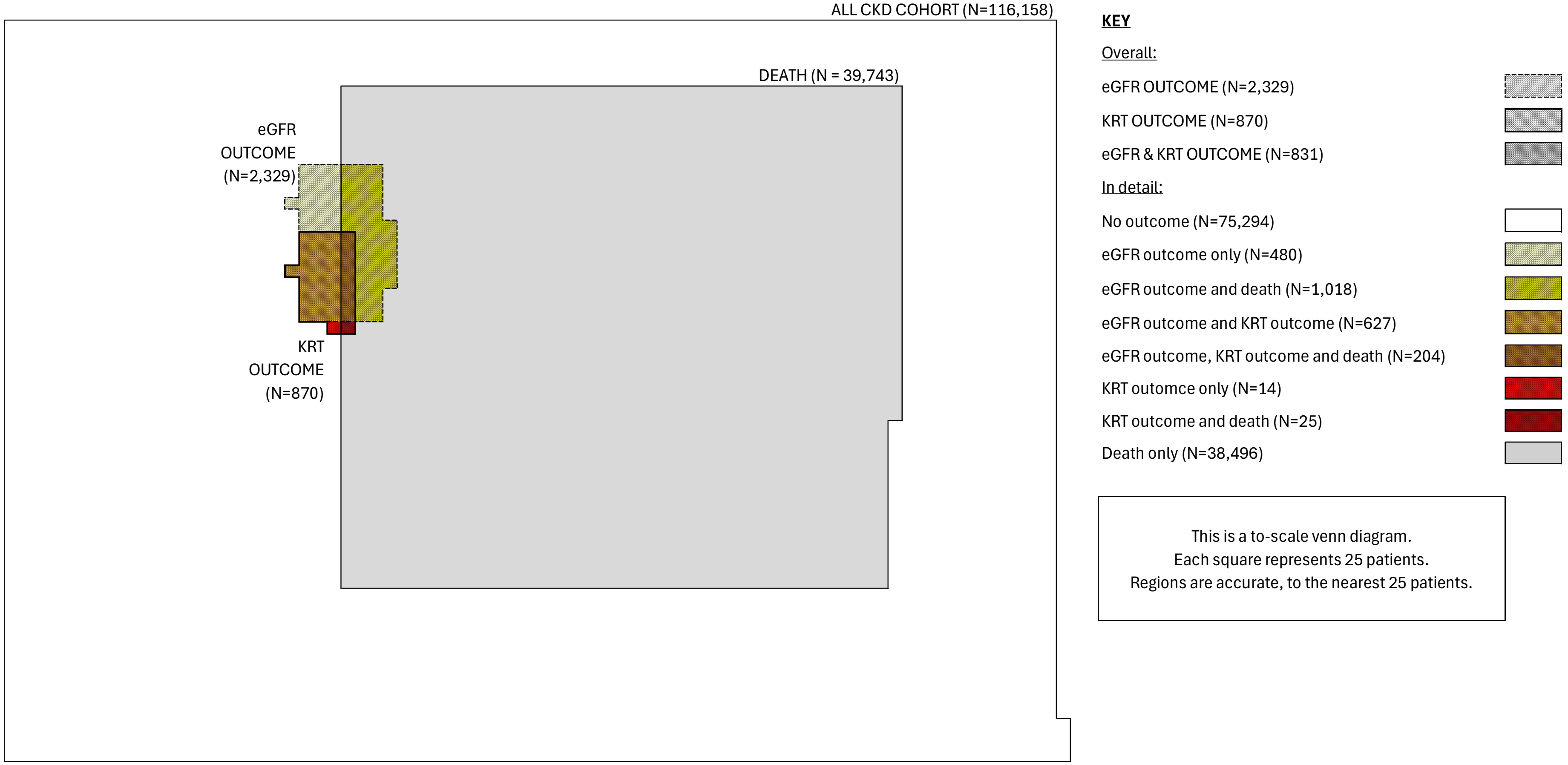

Supplementary Figure 9: Histograms (densities) of linear predictor by outcome status using **new risk equations** in all patients in the entire CKD cohort: (A) KRT, (B) eGFR outcome. Densities are presented for each group (outcome, no outcome) and are overlaid in one plot. Densities should not be confused with frequencies (outcome events appear over inflated compared to non-outcomes, due to rare events). Note that some patients without outcomes are censored before 5 years.

(A)

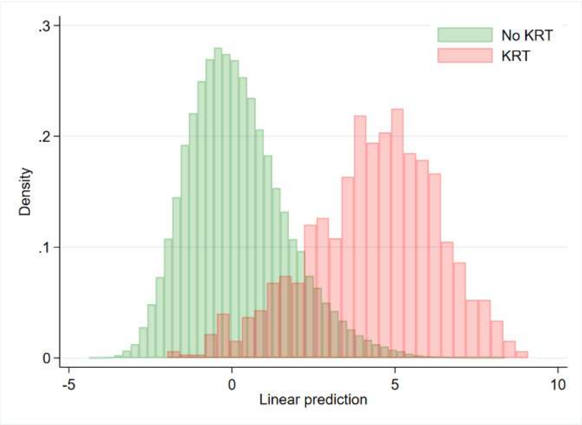

(B)

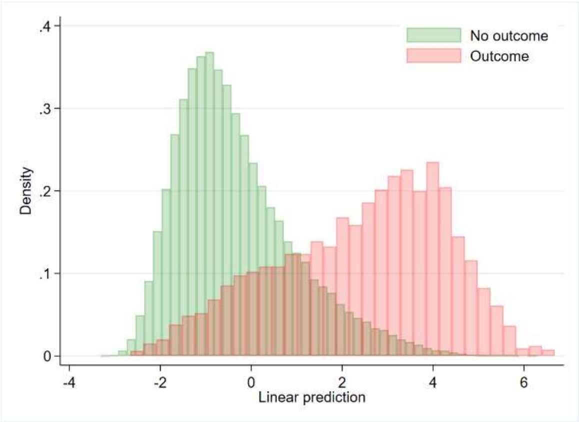

Supplementary Figure 10: Calibration graphics, by subgroup (in entire cohort).
Subgroup plots are shown in the entire cohort, rather than validation cohort, for sample size reasons, and the interest being in heterogeneity in model performance. The all patients plot is shown for completeness, in which case calibration is expected to be almost perfect (overall), with calibration slope close to 1.

KRT:

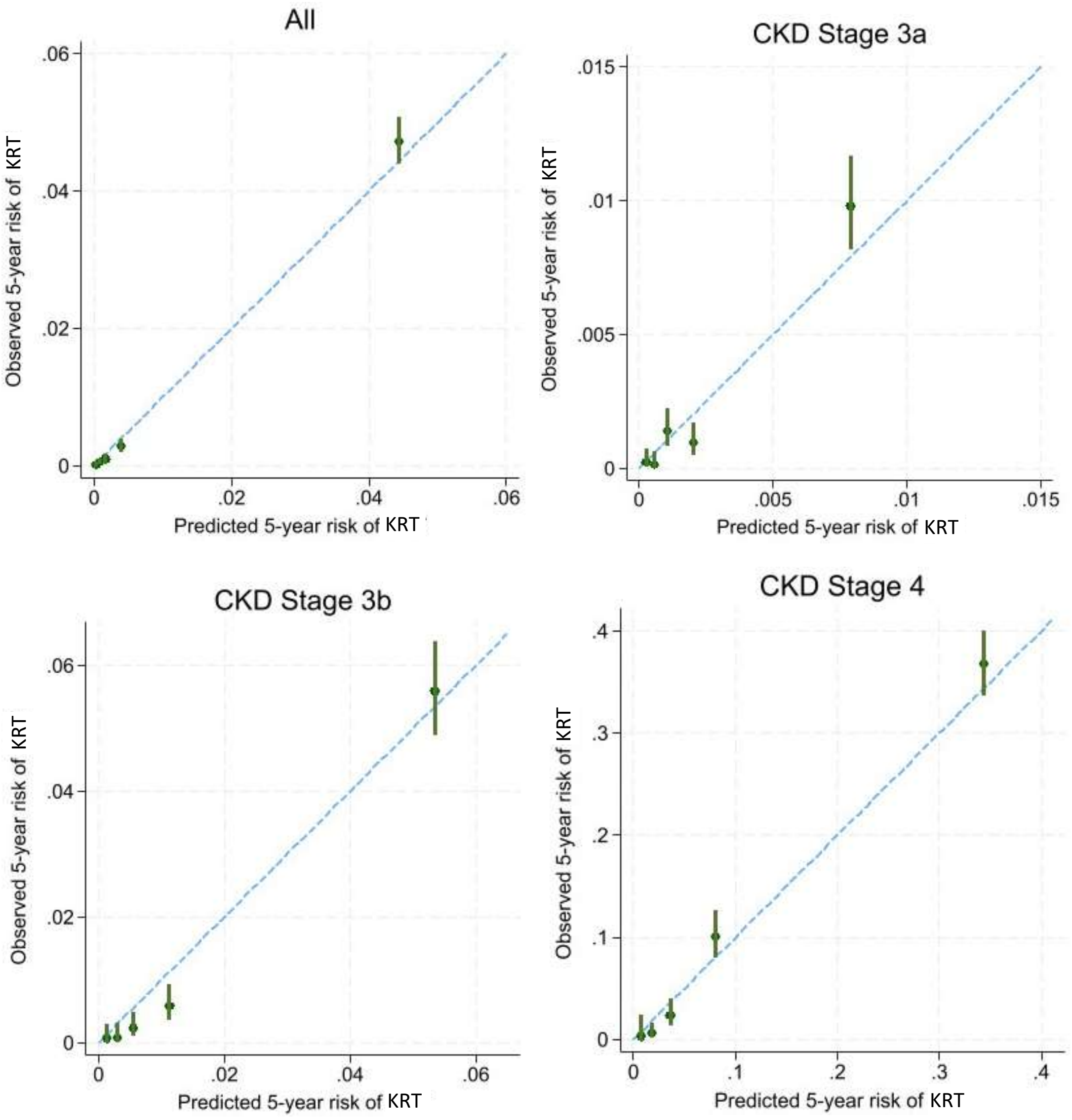

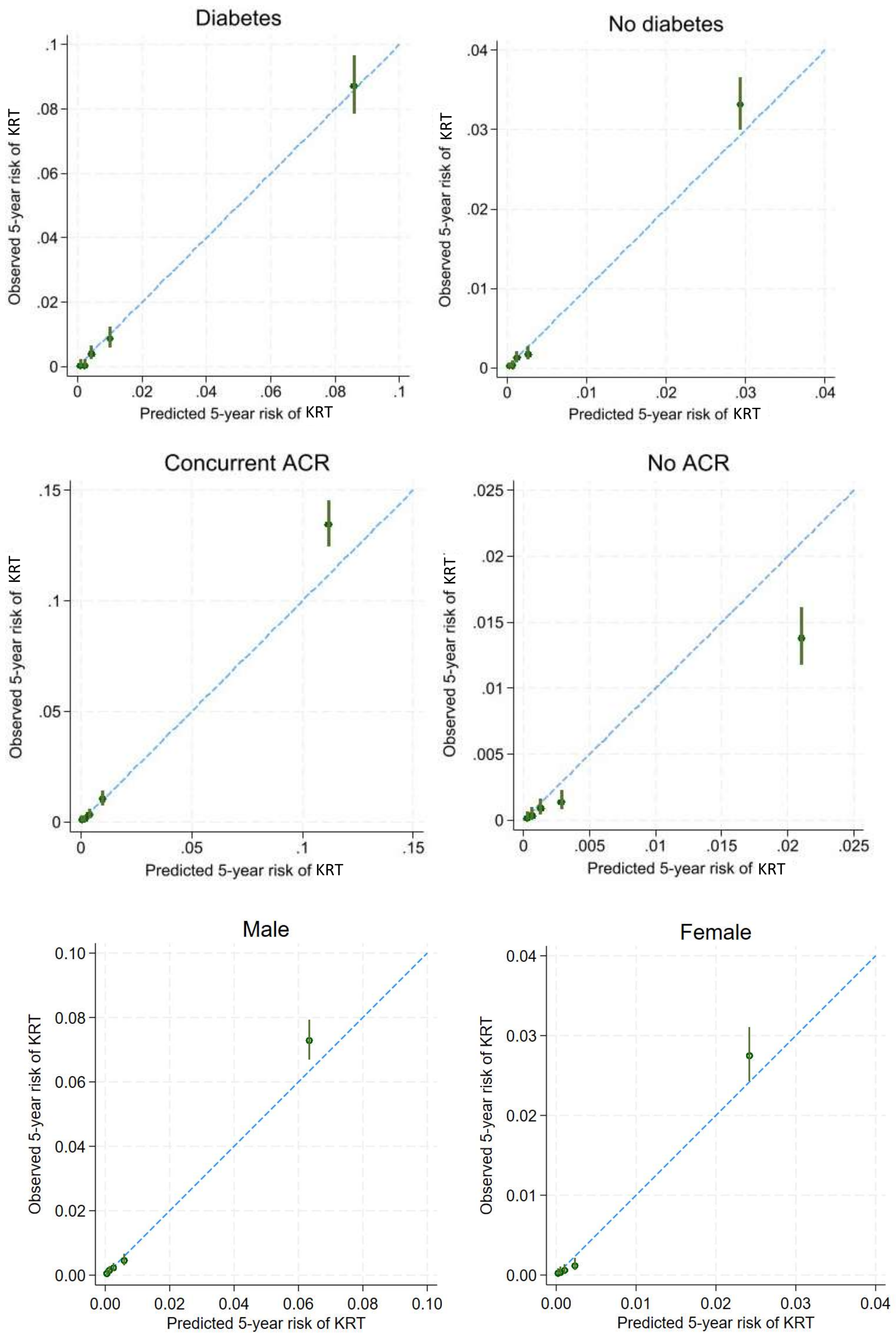
Diabetes
Observed 5-year risk of KRT
Predicted 5-year risk of KRT
No diabetes
Observed 5-year risk of KRT
Predicted 5-year risk of KRT
Concurrent ACR
Observed 5-year risk of KRT
Predicted 5-year risk of KRT
No ACR
Observed 5-year risk of KRT
Predicted 5-year risk of KRT
Male
Observed 5-year risk of KRT
Predicted 5-year risk of KRT
Female
Observed 5-year risk of KRT
Predicted 5-year risk of KRT

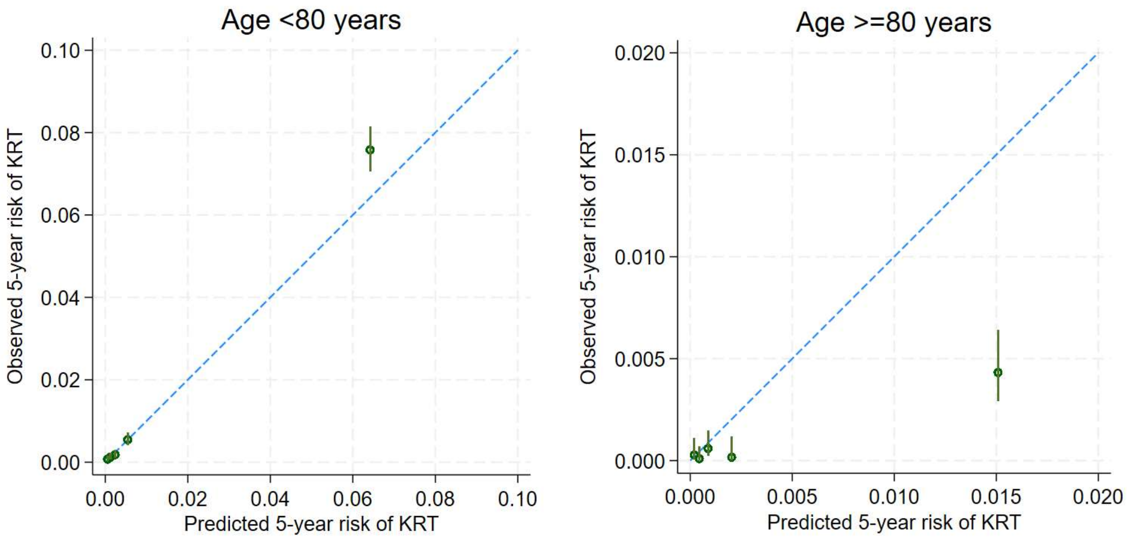

eGFR outcome:

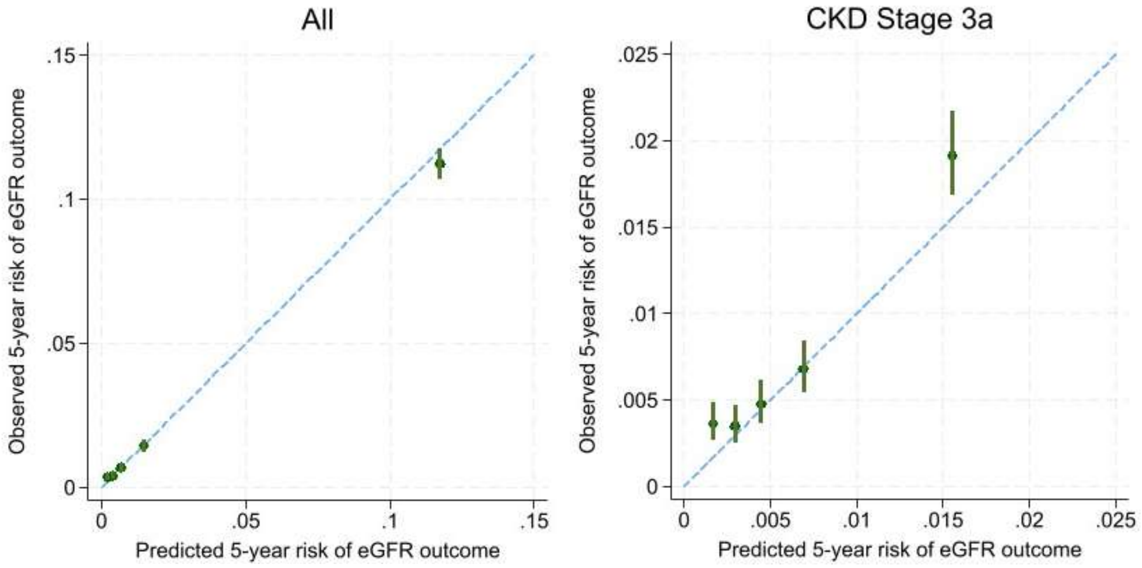

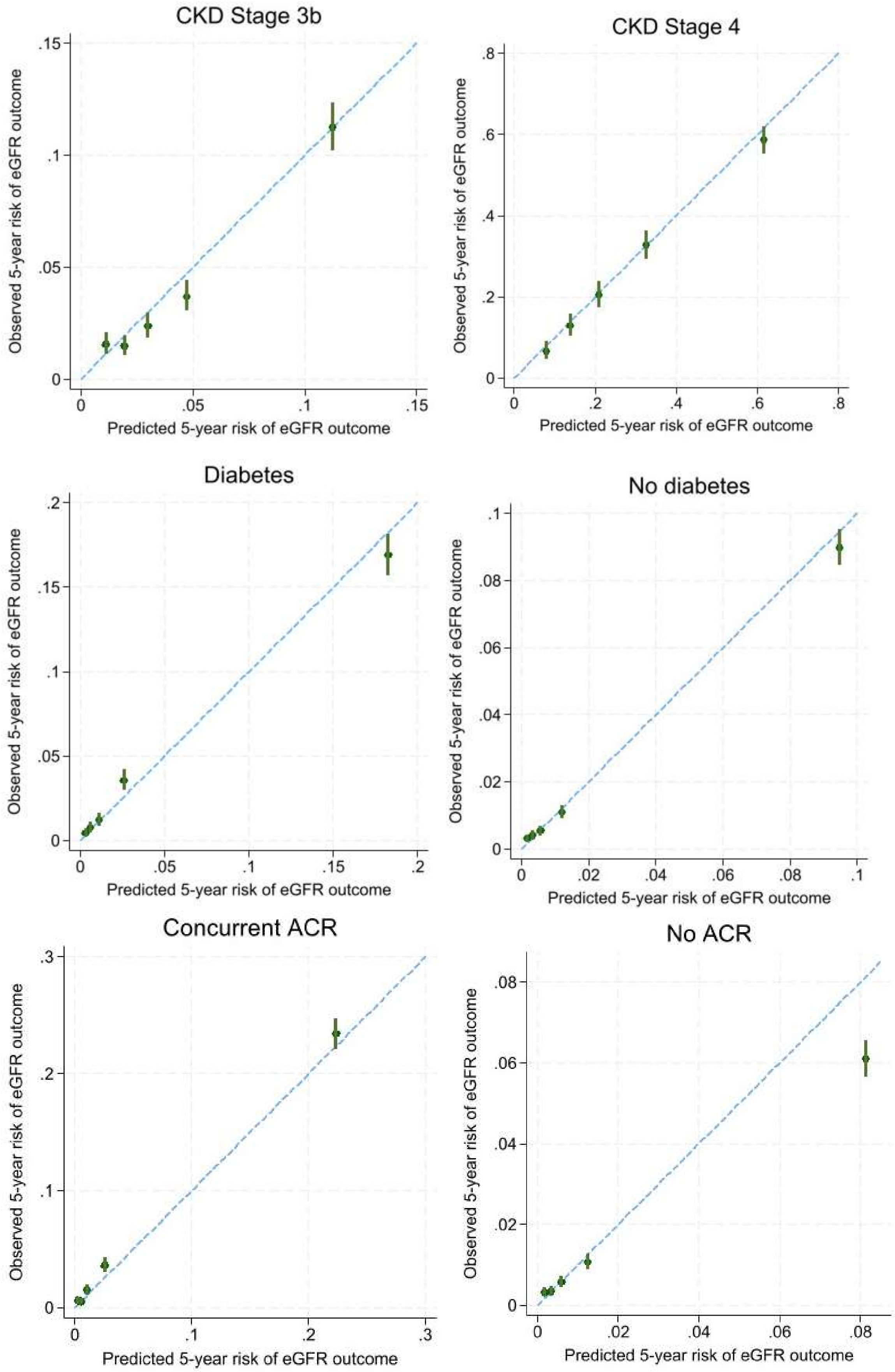
CKD Stage 3b
Observed 5-year risk of eGFR outcome
Predicted 5-year risk of eGFR outcome
0
.05
.1
.15
CKD Stage 4
Observed 5-year risk of eGFR outcome
Predicted 5-year risk of eGFR outcome
0
.2
.4
.6
.8
Diabetes
Observed 5-year risk of eGFR outcome
Predicted 5-year risk of eGFR outcome
0
.05
.1
.15
.2
No diabetes
Observed 5-year risk of eGFR outcome
Predicted 5-year risk of eGFR outcome
0
.02
.04
.06
.08
.1
Concurrent ACR
Observed 5-year risk of eGFR outcome
Predicted 5-year risk of eGFR outcome
0
.1
.2
.3
No ACR
Observed 5-year risk of eGFR outcome
Predicted 5-year risk of eGFR outcome
0
.02
.04
.06
.08

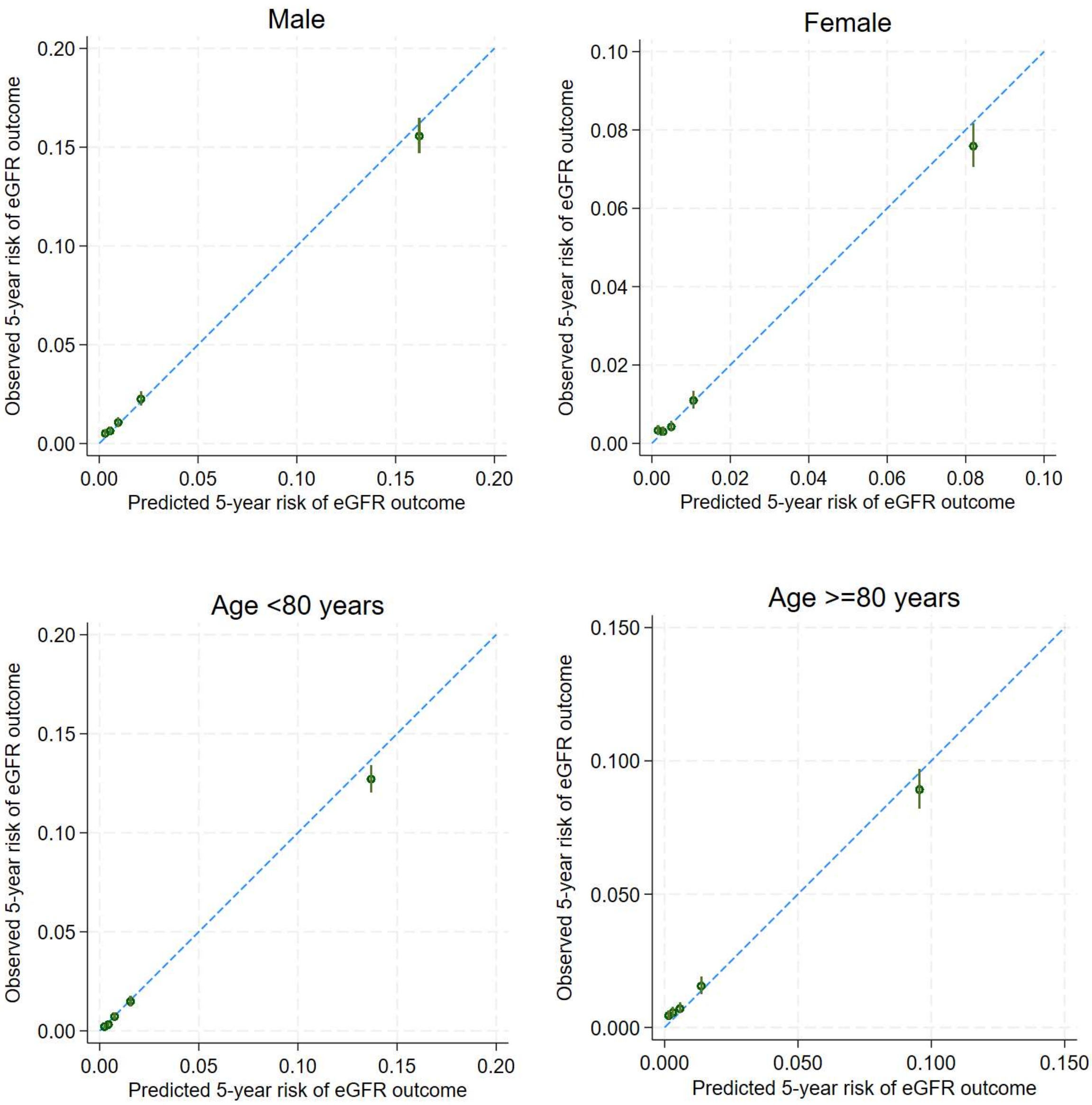
Male
Female
Age <80 years
Age >=80 years
Observed 5-year risk of eGFR outcome
Predicted 5-year risk of eGFR outcome
0.00
0.05
0.10
0.15
0.20
0.02
0.04
0.06
0.08
0.000
0.050
0.100
0.150

Supplementary Figure 11: Observed vs predicted probability of composite outcome non-rebounding eGFR<15 or KRT at 5 years, by quintile of predicted risk, in validation cohort. Quintiles of predicted risk: 0% - 0.246%; 0.246% - 0.398%; 0.398% - 0.654%; 0.654% - 1.357%; 1.357% - 100%.

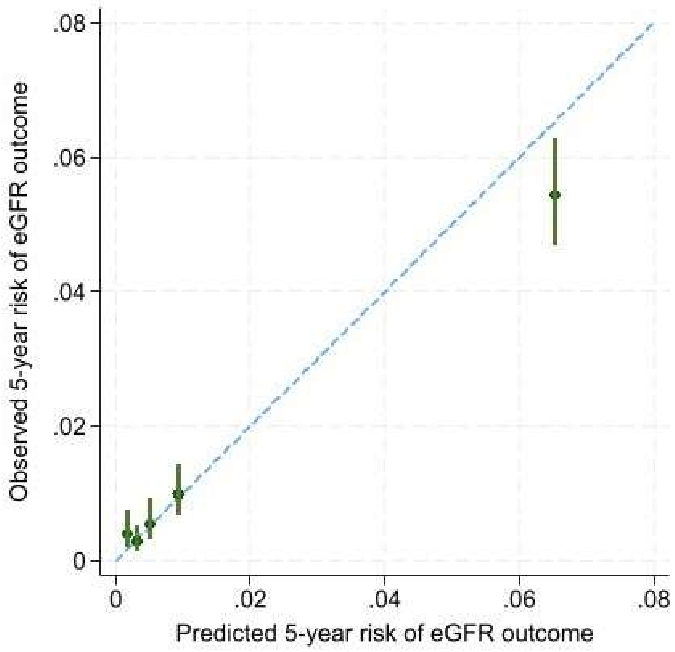

Supplementary Figure 12: Calibration plots for estimation of risks using the regional calibrated 4-variable KFRE in all patients with uACR data: (A) All patients, 5 quintiles; (B) Subset of first 4 quintiles. (Predicted risk quintiles: 0.01% - 0.07%, 0.07% - 0.14%, 0.14% - 0.30%, 0.30% - 1.07%, 1.07% - 95.3%)

(A) **(B)**

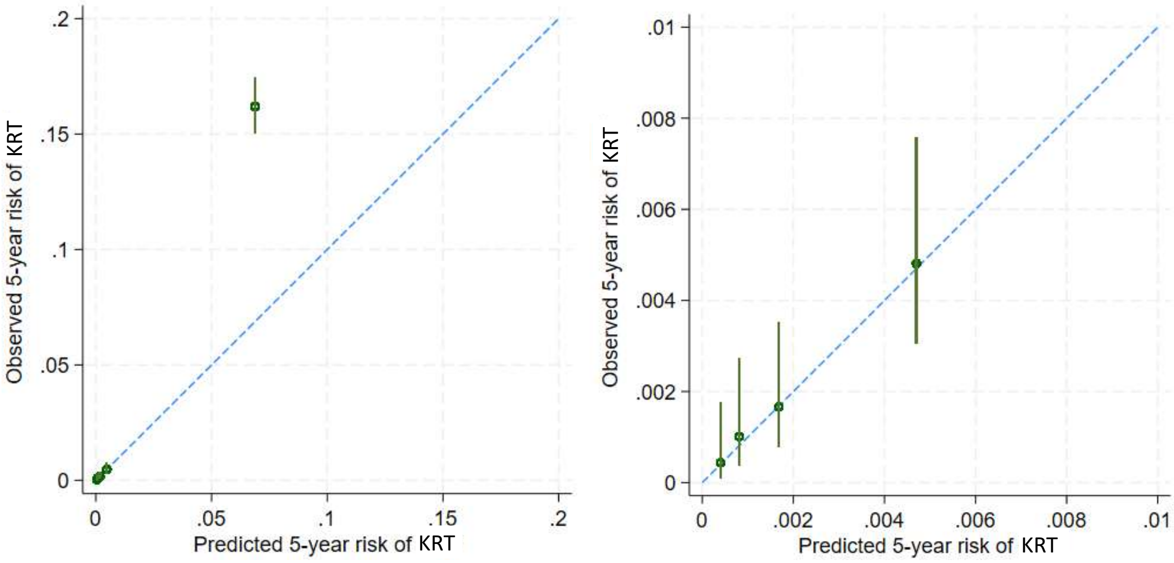

Supplementary Figure 13: Overlaid histograms (densities, not frequencies) of linear predictor by KRT outcome status in all patients in the entire cohort with an uACR result available: (A) 4-variable KFRE; (B) New equation

(A) **(B)**

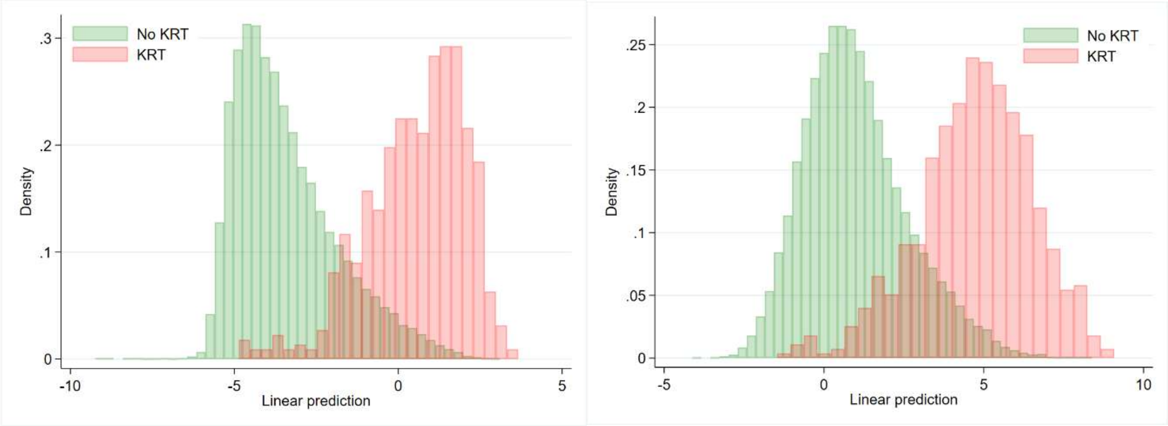

Supplementary Figure 14: Histograms of predicted risks by outcome status, in entire CKD cohort: (A) KRT; (B) non-rebounding eGFR<15 or KRT

(A)

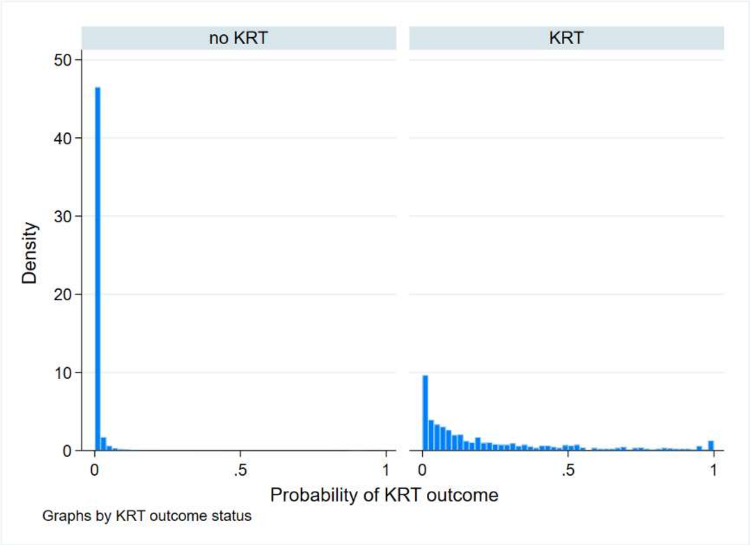

(B)

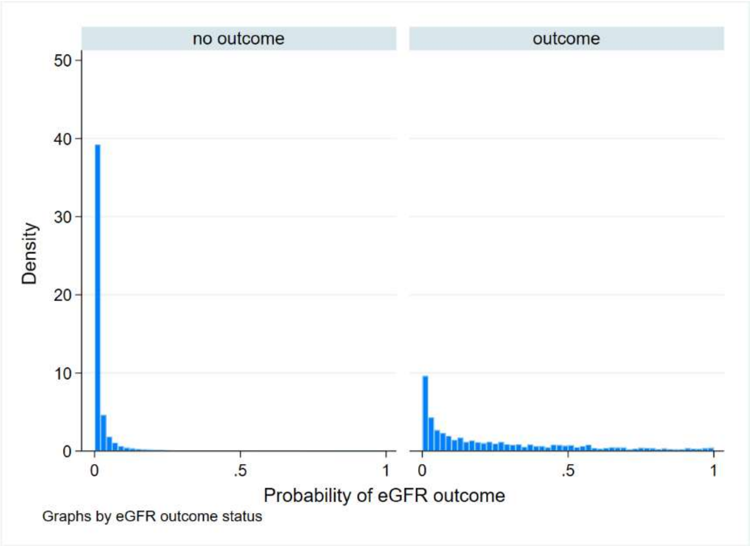

Supplementary Figure 15: Scatter plots comparing (A) predicted risks, (B) linear predictions and (C) ranks of linear predictions, by equation, in patients with uACR data

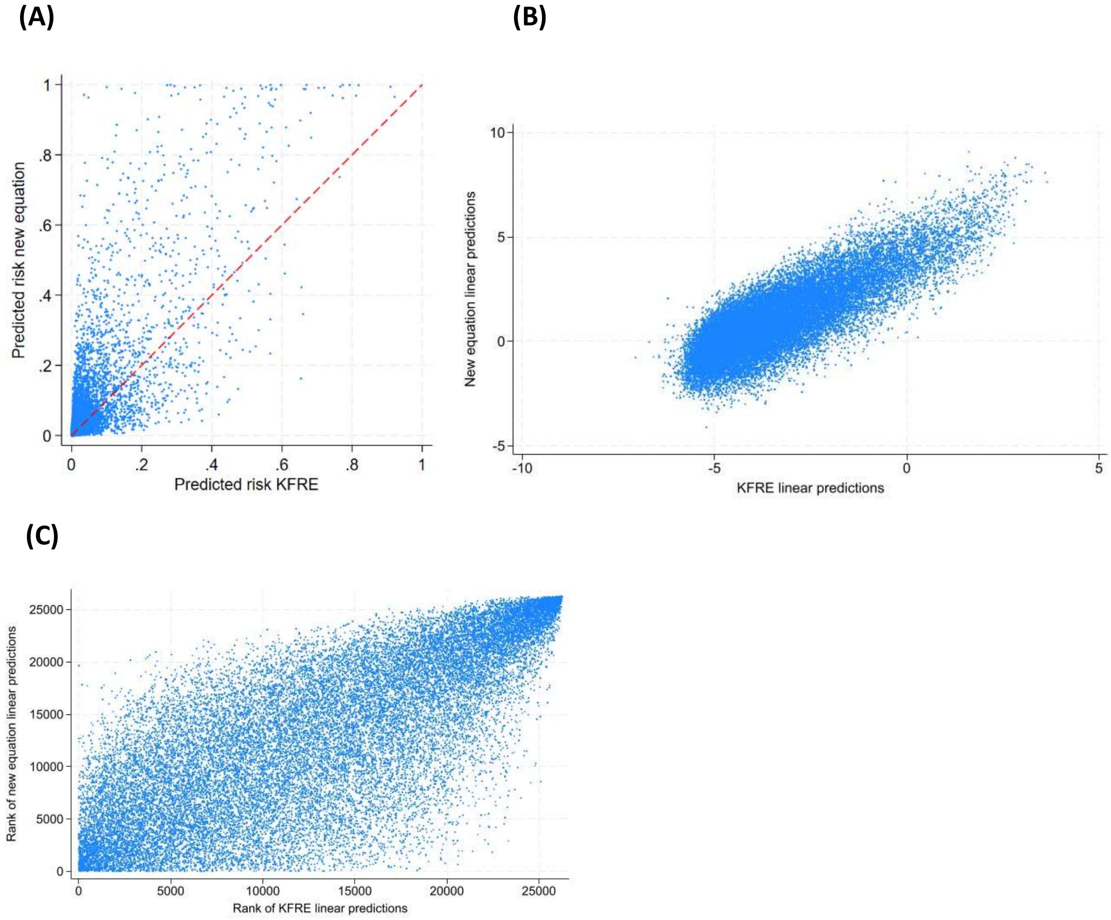

Supplementary Figure 16: Percent shift in rank of linear prediction score, comparing new equation with 4-variable KFRE, in all patients in CKD cohort with an uACR result: (A) All patients; (B) Stratified by KRT outcome status

(A)

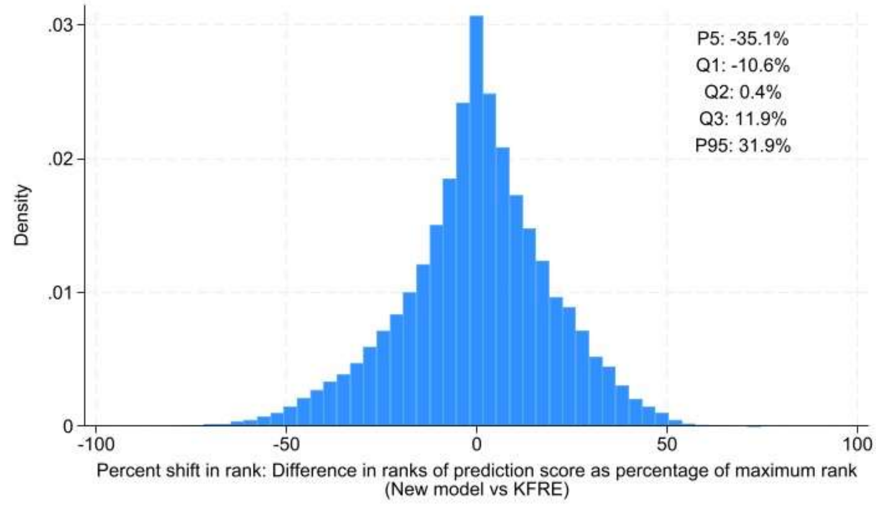

(B)

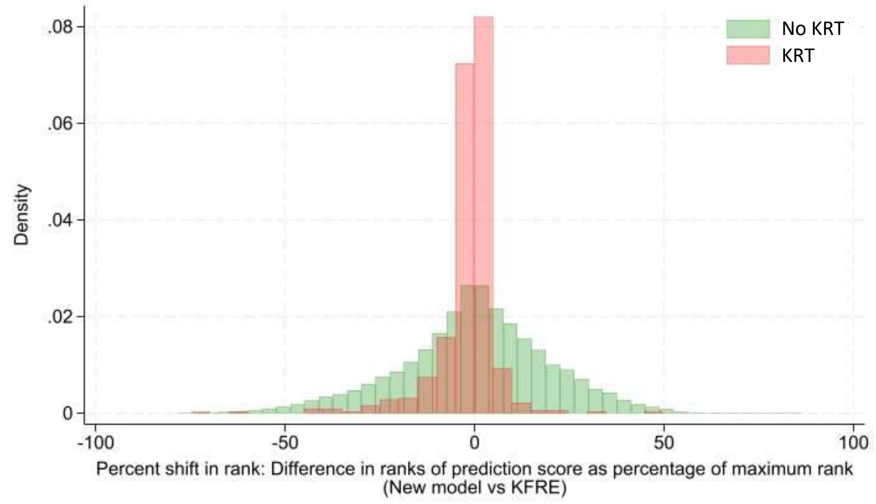

Supplementary Figure 17: Histogram of difference in predicted risks of KRT comparing new equation with KFRE

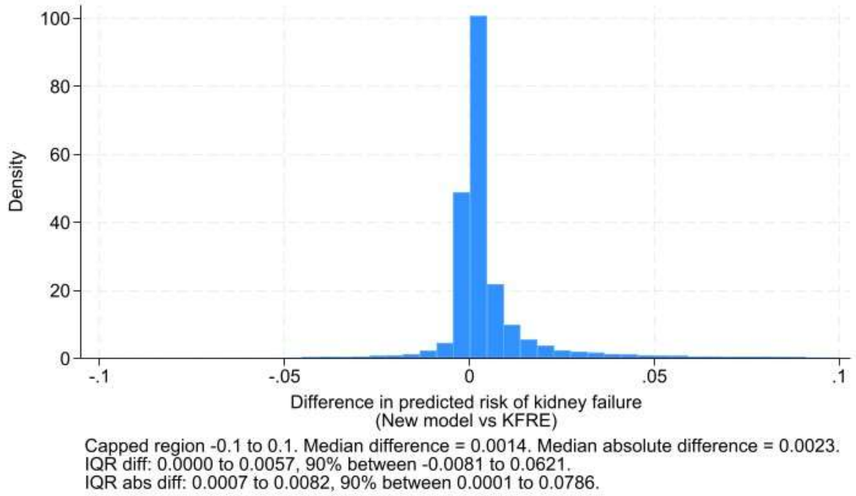

Supplementary Figure 18: Bland-Altman plots demonstrating agreement in logit-transformed predicted probabilities (approximately normally distributed) between the new model for KRT compared to KFRE, with overlaid regression line. To support visualisation, the bottom 0.5% and top 0.5% of observations have been removed.

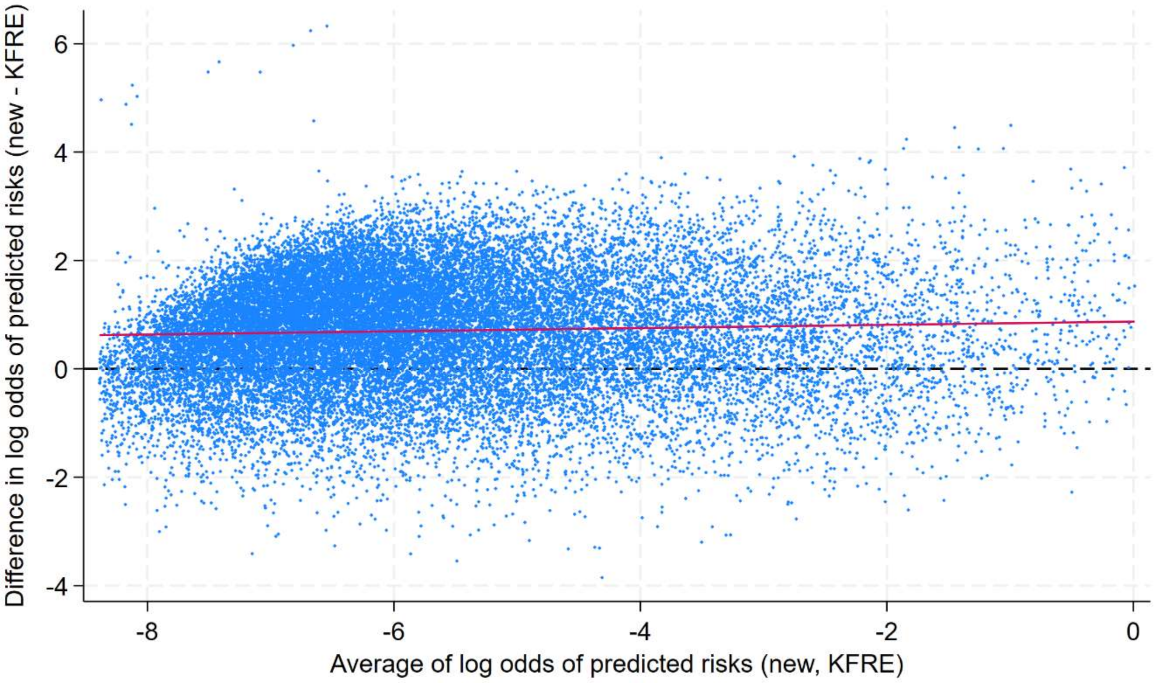

Note that we have plotted for the logit-transformed predicted probabilities as opposed to the linear predictions, due to the fact that the value of both the linear predictor and baseline hazard estimation at 5 years impact estimation of risks (see supplementary information 1 and 2). Since baseline hazard estimations differ between our model and KFRE, this means that comparison of linear predictions alone would not be a fair comparison when assessing how the 2 models agree on estimation of risks. By using this correction, we can readily assess the bias by the density of observations above and below the line y=0 (showing systematic under-estimation of KFRE in this case due to mis-calibration), as well as agreement across the prediction range (no distinctive patterns observed, except perhaps slightly lower variance in distribution for lower predicted risks).

Supplementary Figure 19: Net benefits derived from a decision curve analysis, summarising the excess of clinical benefits compared to expected costs of different decision strategies, namely: treat all (offer further care to all patients regardless of individual risk factors); treat none (do not offer further care to any patients regardless of individual risk factors); new model (offer further care if new model patient predicted risk exceeds desired threshold); KFRE (offer further care if KFRE model patient predicted risk exceeds desired threshold). The most clinically useful model is that with highest net benefit, given desired threshold probability for providing further care. Scale reflects hypothetical risk thresholds between 0-10%.

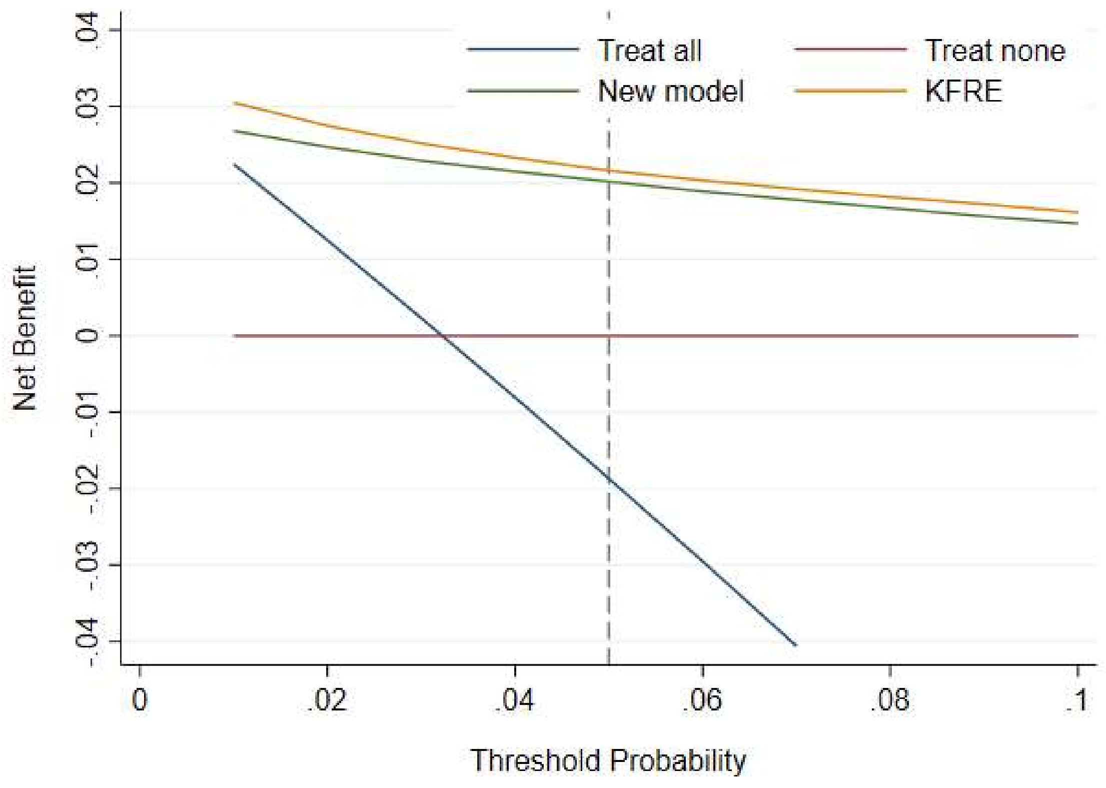

At a threshold of 5% risk of KRT (reflecting the NICE threshold for nephrology referral), the difference in net benefit between KFRE and our new equation in the population of patients with uACR data is 0.00124. This is interpreted as the increase in proportion of true positives (predicted risk > 5% and KRT occurred) without a change in false positives (predicted risk > 5% but no KRT) when using KFRE instead of our new equation. This translates to a “cost” of using our new equation over KFRE (in patients typically tested for uACR) of: 1 *fewer* patients escalated for further care who *will* go on to develop kidney failure per 806 patients evaluated (or equivalently, 1 *additional* patient escalated for further care who *will not* go on to develop kidney failure per 42 patients evaluated).

Supplementary Information 1: Details of new prediction equations

The following details show how we arrive at an equation for risk of kidney failure within 5 years of patient identification using results of the final Cox regression model.

Probability of survival at time t years, $\hat{S}_i(t)$, is estimated using the probability of survival at time t years in the baseline group, $\hat{S}_0(t)$, and the value of the linear predictor for an individual patient, $\boldsymbol{x}_i\widehat{\boldsymbol{\beta}}$, as follows:

$$\hat{S}_i(t) = \hat{S}_0(t)^{\exp(\boldsymbol{x}_i\widehat{\boldsymbol{\beta}})}$$

The baseline survival function, $\hat{S}_0(t)$, is estimated from the analysis dataset. We estimate the result at 5 years for KRT outcome as $\hat{S}_0(t = 5\ years)$ = 0.99839.

Coefficient estimates $\widehat{\boldsymbol{\beta}}$ are the log(hazard ratio) estimates for each covariate from the regression model (presented as hazard ratio estimates in Table 2).

The final prediction equation is as follows:

P(RRT within 5 years of identification) = 1 – 0.99839 x exp{
(-0.64373) x ((baseline eGFR – 47.851)/5)
+ (-0.71971) x ((age – 78.287)/10)
+ (0.76776) x (1 if diabetes, 0 otherwise)
+ (-0.61870) x (1 if female, 0 if male)
+ (-0.57871) x (1 if AF, 0 otherwise)
+ (0.48235) x (1 if ACEiARB, 0 otherwise)
+ (0.40786) x (1 if PAD, 0 otherwise)
+ (0.034136) x ((eGFR decline per year – 3.3343)/5)
+ (-0.56595) x (1 if AKI, 0 otherwise)
+ (0.23353) x (1 if hypertension, 0 otherwise)
}

For precise details of variable definitions, see methods section.

Similarly, the final prediction equation for eGFR outcome is:

P(non-rebounding eGFR<15 or RRT within 5 years of identification) = 1 – 0.98717 x exp{
(-0.70161) x ((baseline eGFR – 47.851)/5)
+ (-0.36603) x ((age – 78.287)/10)
+ (-0.58579) x (1 if female, 0 if male)
+ (0.37122) x (1 if diabetes, 0 otherwise)
+ (0.22808) x (1 if PAD, 0 otherwise)
+ (0.12733) x (1 if HF, 0 otherwise)
+ (-0.17506) x (1 if CHD, 0 otherwise)
+ (-0.24789) x (1 if AF, 0 otherwise)
+ (-0.33448) x (1 if AKI, 0 otherwise)
}

An alternative version of the final model for KRT, with AKI variable removed is as follows:

P(RRT within 5 years of identification) = 1 – 0.99843 x exp{
(-0.64108) x ((baseline eGFR – 47.851)/5)
+ (-0.72100) x ((age – 78.287)/10)
+ (0.76777) x (1 if diabetes, 0 otherwise)
+ (-0.60854) x (1 if female, 0 if male)
+ (-0.58355) x (1 if AF, 0 otherwise)
+ (0.48786) x (1 if ACEiARB, 0 otherwise)
+ (0.41054) x (1 if PAD, 0 otherwise)
+ (0.02948) x ((eGFR decline per year – 3.3343)/5)
+ (0.24432) x (1 if hypertension, 0 otherwise)
}

Supplementary Information 2. Equation for 4-variable KFRE

The regional calibrated 4-variable KFRE (non-North America) for predicting 5 year risk was used in model comparisons, as follows:

$$\text{Predicted risk} = 1 - 0.9365 \wedge \exp(-0.2201 \times (\text{age}/10 - 7.036) + 0.2467 \times (\text{male} - 0.5642) - 0.5567 \times (\text{eGFR}/5 - 7.222) + 0.4510 \times (\log(\text{uACR}) - 5.137))$$
