# Supplementary material for: Developing a new albuminuria-free risk prediction equation for kidney failure in patients with chronic kidney disease: retrospective cohort study
Source: BMJ Med. 2026 Feb 2;5(1):e001950. doi: 10.1136/bmjmed-2025-001950 (PMC12878267; doi:10.1136/bmjmed-2025-001950)
Supplement: Supplementary file 3 [file bmjmed-2025-001950.reviewer_comments.pdf]

PEER REVIEW HISTORY

BMJ Medicine publishes all reviews undertaken for accepted manuscripts. Reviewers are asked to complete a checklist review form and are provided with free text boxes to elaborate on their assessment. These free text comments are reproduced below.

ARTICLE DETAILS

**TITLE (PROVISIONAL)**	Developing a new albuminuria-free risk prediction equation for kidney failure in patients with chronic kidney disease: retrospective cohort study
**AUTHORS**	Cleary, Faye; Prieto Merino, Antonio David; Major, Rupert; Carrero, Juan; Nitsch, Dorothea

VERSION 1 - REVIEW

**REVIEWER NAME**	Ellison, Isabella
**REVIEWER AFFILIATION**	
**REVIEWER CONFLICT OF INTEREST**	None
**AI DISCLOSURE**	No
**DATE REVIEW RETURNED**	18-Aug-2025

**GENERAL COMMENTS**	Clear to follow. Large sample size and appreciated the subgroup analysis. Good long term follow up

**REVIEWER NAME**	Lees, Jennifer
**REVIEWER AFFILIATION**	University of Glasgow, Institute of Cardiovascular and Medical Sciences
**REVIEWER CONFLICT OF INTEREST**	None
**AI DISCLOSURE**	No
**DATE REVIEW RETURNED**	22-Aug-2025

**GENERAL COMMENTS**	The authors have used linked data, representative of the general population managed in routine care in Stockholm, Sweden, from the SCREAM resource. The authors have created a new risk prediction tool for use among patients with CKD stage 3-4 (eGFR 30-60 mL/min/1.73m2) to predict kidney failure using data routinely available in the clinical record, and without using albuminuria. The authors found that their new tool showed high discrimination and good calibration, with only slightly lower discrimination and slightly lower performance on decision curve analysis than the widely used and recommended 4-variable KFRE. Given the lack of widespread testing of albuminuria, it is welcome to know that there may be a clinically useful alternative.<br><br>The manuscript is generally well written. The analysis is appropriate and comprehensive. The addition of subgroups is welcome. The new score is potentially useful in clinical practice.<br><br>I do have a few (mostly minor) comments and queries, primarily aiming to improve clarity. I think some of these clarifications would

	be helpful for the generalist audience of BMJ Medicine:<br><br>1. Introduction (lines 20-26): the authors briefly discuss KFRE. For the non-expert, I would suggest specifying here that the KFRE is for use in people with pre-existing CKD (by eGFR criteria) and that it is used to predict 2- and 5- year risk of kidney failure and perhaps mention risk thresholds that might warrant further action (accepting that these vary by indication and guideline).<br>2. Methods / data sources: the 2nd and 3rd sentences start with “it”. Suggest changing “it” to specify “SCREAM”.<br>3. Methods: The description of use of 2009 CKD-EPI formula seems out of place in the “data sources” section. Suggest move this description to the “predictors” section of methods.<br>4. Methods: The authors have appropriately matched the eligible population for their new risk score with the population in which KFRE was developed and validated. I suggest making this more explicit in the methods (e.g. in section on “participants”).<br>5. Methods / predictors: since there is excellent access to prescribed medication data in SCREAM, why did the authors not also include prescription of other medications that may be indicative of relevant comorbidities as well as ACE/ARB, e.g. antiplatelet as indicator of vascular disease, inhalers as indicator of obstructive airways disease, other antihypertensives as indicators of hypertension? I imagine this is because ACE/ARB are probably more likely to select people at high risk of albuminuria. If so, suggest specifying this in the methods.<br>6. Methods / missing data: for those unfamiliar with eGFR slope, suggest adding just a few words about why eGFR slope was missing in 2.4% patients (fewer than 3 historical eGFR results). This may not be entirely clear no those unfamiliar with eGFR slope, in part because predictors section does not mention eGFR slope but describes this instead as “prior annual eGFR decline”.<br>7. Methods / statistical analysis: Briefly mention why modelling was capped at 5 years pots-index date (presumably to match 5-year maximum prediction horizon as in KFRE).<br>8. Methods / statistical analysis: at end of the 1st paragraph in this section, it is stated “all estimation methods account for censoring” – please specify what methods of censoring here. In the paragraph below it mentions censoring for death but would make this more explicit. Perhaps also specify here that the model does not account for death as a competing risk (as for KFRE).<br>9. Results / model performance: 2nd paragraph in this section: “Model discrimination was good for the sensitivity outcome (Table 2) but inferior to KRT analysis.” – I think it would be clearer to specify what the sensitivity outcome is here, and change “KRT analysis” to “prediction of first initiation of KRT”.<br>10. Since there are differences in observed likelihood of kidney failure by sex (as well as diabetes status, age), I would be interested to know if there was any difference in performance of this new equation (and KFRE) in sex subgroups.<br>11. In my opinion, the manuscript could advocate more strongly for better testing for albuminuria, as this remains an important marker of risk and response to treatment even beyond its utility in the KFRE.<br>12. Do the authors plan to share analysis code?<br>13. Have the authors considered creating e.g. a shiny app for their new risk score?

**REVIEWER NAME**	Bentall, Andrew John
**REVIEWER AFFILIATION**	Mayo Clinic, Division of Nephrology

REVIEWER CONFLICT OF INTEREST	None
AI DISCLOSURE	No
DATE REVIEW RETURNED	09-Sep-2025

| GENERAL COMMENTS | The authors set out to use the Stockholm database for patients without urinary albumin using clinical risk factors for the development of End stage kidney disease as an alternative to KFRE equation. Development population was 85,000 and the validation cohort was 28,000. There were 854 events across both groups. 0.8% in the development cohort and 0.4% in the validation cohort.<br><br>Why were the differences in KRT used rather than eGFR <15ml/min which could increase endpoint number? I see in the discussion and figures use of eGFR as an endpoint but do not see the methodology in the paper of this described.<br>The SCREAM population is almost 50% of the population were over 80 years old and the majority of the population had CKD stage 3a 45-60ml/min. The authors should determine the proportion of patients in each age group with each different GFR and endpoint. Whilst there was discussion on proportional analysis which would help to predict end stage kidney disease, death without dialysis is also an important endpoint.<br>The authors describe the data of those with higher risk of KTR being younger in Supplemental Table 6.<br><br>Given that this study was submitted in 2025, the eGFR equation should use the CKD EPI 2021 formula, as it will also apply better to other populations if used going forward. There is likely little numeric difference particularly in a Scandinavian cohort, but is more contemporaneous to current practices. No ethnicity is reported in the Table 1 demographics.<br><br>Using historical eGFR linear regression to demonstrate eGFR decline in prior 3 years is hard value to use in clinical practice – requires increased data that may not be present in the majority of patients.<br><br>in epidemiological studies difficult to determine, but<br>GFR<15 – described in starting KRT – 25% died, but those not starting 68% died. This is likely the result of end of life conversations and thus KRT might not be the best endpoint for sensitivity. Understanding the differences in these populations would help the reader.<br><br>The risk models demonstrate that the younger the patient (median age 64-67) are higher risk of reaching KTR stage. Supporting the fact that >80yrs old with CKD stage 3a are not at significant risk of progression.<br><br>Supplementary Figure 18 shows that using KFRE is still better than new model at 5% risk per NICE guidelines.<br><br>Whilst the 3 variable model does not perform as well as the full model, it would be easier to implement in PCP for screening for CKD progression than the full model.0.936 vs. 0.944 is excellent model prediction value.<br><br>In summary, the model performs well statistically but the number of |

	variables would be hard to implement in clinical practice. There is not a significant improvement on the validation of the KFRE equation, but a simpler 3 riskf actor equation could improve risk stratification in the population and lead to further testing including urinary albumin where a more accurate KFRE formula could then be applied.<br><br>Questions<br>1. Authors use the UK system as an introduction, but the study is performed in a Swedish cohort with different healthcare systems. Populations may or may not be different given immigration, multi-ethnic UK population and increased CKD in non-Caucasian UK population. Hard to directly extrapolate.<br>2. Given that the lead authors are from the UK, could the validation have been done in a UK primary care database? The age of the population in the SCREAM database is a potential weakness.

**REVIEWER NAME**	Perera, Rafael
**REVIEWER AFFILIATION**	University of Oxford, Primary Care Health Sciences
**REVIEWER CONFLICT OF INTEREST**	None
**AI DISCLOSURE**	I have used Grammarly to help with spelling and grammar when editing my review.
**DATE REVIEW RETURNED**	30-Sep-2025

**GENERAL COMMENTS**	bmjmed-2025-001950: Developing a new albuminuria-free risk prediction equation<br>for kidney failure in patients with chronic kidney disease: retrospective cohort study<br><br>Stats Review:<br><br>The present manuscript provides results from a study that developed and internally validated a risk prediction model for kidney failure (KRT – Kidney Replacement Therapy) and a sensitivity outcome of 'non-rebounding eGFR<15 or KRT. Crucially, this risk prediction model does not include albumin:creatinine ratio (uACR) as the recording of this biomarker is only available for a minority of patients with CKD. This derivation and validation utilise data from over 116,000 participants in Sweden (the Stockholm Creatinine Measurements (SCREAM) database).<br><br>The manuscript is generally well-written, and the model is likely to be useful in systems with mature healthcare records, as the automatic estimation of these risk scores could be easily employed.<br><br>There are some queries and clarifications about the approach taken and the reporting of the study that would benefit from addressing:<br><br>• A significant proportion of participants died during the study. This is not the primary outcome or part of a composite one. Given this, it is unclear what the impact of this 'competing risk' is on the estimated model. It is unclear whether this would likely create bias or impact the calibration of the risk score. Further analyses taking death into consideration might be required.<br>• Variable selection using 'backward selection' has generally been superseded by regularisation techniques (e.g. Lasso). Please provide a rationale/justification for backward selection.

	• There was a lack of clarity about the selection of the period under study. There is a table that aims to clarify this, but a brief explanation of the rationale is still required (apologies if I have missed this).<br>• The event counts per variable appear to be relatively large and likely sufficient; however, it would be useful to understand whether a formal sample size estimation was used and the parameters associated with it.<br>• A critical analysis comparing those with uACR measured to those without suggests some relevant differences in risk levels. Some of this is managed by the relevant other variables included in the model (beyond those included in KFRE). The authors suggest this might be related to medication practices being used as proxies for uACR. Worth discussing how transportable this specific aspect is to other settings outside Sweden.<br>• Please expand on how the bootstrap resampling was used as part of the first internal validation using the development cohort. Were these used to determine a better overall estimate of the models or for model selection? Please specify. Related to this point, please justify the use of 200 bootstrap samples.<br>• When comparing linear predictors, consider using alternative methods for testing agreement, such as Bland-Altman plots. These could help identify systemic biases within the range of predictions.<br>• For Supplementary Figure 6, the event rates between the development and validation cohorts differ significantly. Could this be explored in the Discussion?

VERSION 1 – AUTHOR RESPONSE

Reviewer 1 (Dr Isabella Ellison) comments	Responses
Clear to follow. Large sample size and appreciated the subgroup analysis. Good long term follow up	Thank you.

Reviewer 2 (Dr. Jennifer Lees, University of Glasgow) comments	Responses
The authors have used linked data, representative of the general population managed in routine care in Stockholm, Sweden, from the SCREAM resource. The authors have created a new risk prediction tool for use among patients with CKD stage 3-4 (eGFR 30-60 mL/min/1.73m2) to predict kidney failure using data routinely available in the clinical record, and without using albuminuria. The authors found that their new tool showed high discrimination and good calibration, with only slightly lower discrimination and slightly lower performance on decision curve analysis than the widely used and recommended 4-variable KFRE. Given the lack of widespread testing of albuminuria, it is welcome to know that there may be a clinically useful alternative.	

The manuscript is generally well written. The analysis is appropriate and comprehensive. The addition of subgroups is welcome. The new score is potentially useful in clinical practice.<br><br>I do have a few (mostly minor) comments and queries, primarily aiming to improve clarity. I think some of these clarifications would be helpful for the generalist audience of BMJ Medicine:<br><br>1. Introduction (lines 20-26): the authors briefly discuss KFRE. For the non-expert, I would suggest specifying here that the KFRE is for use in people with pre-existing CKD (by eGFR criteria) and that it is used to predict 2- and 5-year risk of kidney failure and perhaps mention risk thresholds that might warrant further action (accepting that these vary by indication and guideline).<br><br>2. Methods / data sources: the 2nd and 3rd sentences start with "it". Suggest changing "it" to specify "SCREAM".<br><br>3. Methods: The description of use of 2009 CKD-EPI formula seems out of place in the "data sources" section. Suggest move this description to the "predictors" section of methods.<br><br>4. Methods: The authors have appropriately matched the eligible population for their new risk score with the population in which KFRE was developed and validated. I suggest making this more explicit in the methods (e.g. in section on "participants").<br><br>5. Methods / predictors: since there is excellent access to prescribed medication data in SCREAM, why did the authors not also include prescription of other medications that may be indicative of relevant comorbidities as well as ACE/ARB, e.g. antiplatelet as indicator of vascular disease, inhalers as indicator of obstructive airways disease, other antihypertensives as indicators of hypertension? I imagine this is because ACE/ARB are probably more likely to select people at high risk of albuminuria. If so, suggest specifying this in the methods.<br><br>6. Methods / missing data: for those unfamiliar with eGFR slope, suggest adding just a few	1. Thank you. I have amended the introduction (paragraph 2) to specify that the KFRE is recommended for use in pre-existing CKD with 2 and 5 year equations. I have also highlighted the use of a 5% risk threshold in primary care to indicate referral for specialist care, as detailed in NICE guidelines, which is relevant to this work.<br><br>2. I have removed the word "it", replacing with "SCREAM" on first occurrence and "this data" on second occurrence.<br><br>3. The reason for including details of eGFR estimation rules in the data sources section (as well as which eGFR measures were used in this study) is that it impacts not just predictor estimation but also identification of the study population and outcome estimation (i.e. sections for Participants, Outcomes and Predictors). I therefore would prefer to keep this text where it is.<br><br>4. I have added this clarification to the participants section, stating that KFRE was developed and validated in the population of CKD stages 3-5 prior to any initiation of KRT.<br><br>5. Thank you. Yes, that's exactly right. Since ACEi/ARBs are commonly prescribed in CKD patients with albuminuria, we anticipated that ACEi/ARB use could provide a strong proxy for albuminuria, in the absence of regular uACR testing, and hence be potentially highly predictive of kidney failure risk. I have added a sentence on this to the methods-predictors section. In general, candidate predictor selection was based on pragmatic decisions in clinical team discussions to include variables considered most likely to be predictive of kidney failure risk using data that was likely to be routinely available.<br><br>6. To improve clarity throughout document, I have amended occurrences of text on "eGFR slopes" to "eGFR slopes of decline". I have also amended text in the methods-missing data section to state that the reason for missing slopes in some patients was fewer than 3 historical eGFR results.<br><br>7. Yes, exactly, follow up was capped at 5 years due to the intention to predict risks within the next 5 years. I have added a few words to the methods-statistical analysis section to clarify this. Follow-up duration available in the dataset ranged between patients from 3 years up to

words about why eGFR slope was missing in 2.4% patients (fewer than 3 historical eGFR results). This may not be entirely clear no those unfamiliar with eGFR slope, in part because predictors section does not mention eGFR slope but describes this instead as “prior annual eGFR decline”.	beyond 10 years in some patients. While including longer follow-up could have improved analytical power, it would require an assumption that hazard ratios for kidney failure estimated in a 5-year window would continue to hold over longer durations. Such an assumption could be easily violated, and was not necessary in our study which already had very good power.
7. Methods / statistical analysis: Briefly mention why modelling was capped at 5 years pots-index date (presumably to match 5-year maximum prediction horizon as in KFRE).	
8. Methods / statistical analysis: at end of the 1st paragraph in this section, it is stated “all estimation methods account for censoring” – please specify what methods of censoring here. In the paragraph below it mentions censoring for death but would make this more explicit. Perhaps also specify here that the model does not account for death as a competing risk (as for KFRE).	8. Ok, thanks. The sentence “All estimation methods account for censoring.” refers to the evaluation of model discrimination. I have amended this sentence to “Methods for evaluation of model discrimination account for censoring for death and end of follow up occurring prior to event occurrence.” I have also specified that censoring for death is the same approach as KFRE, and estimated risks do not account for the competing risk of death, as suggested. This means that estimated risks over the 5-year horizon assume that death will not occur during this time, and that the risk of death is independent of the risk of kidney failure, which I have clarified.
9. Results / model performance: 2nd paragraph in this section: “Model discrimination was good for the sensitivity outcome (Table 2) but inferior to KRT analysis.” – I think it would be clearer to specify what the sensitivity outcome is here, and change “KRT analysis” to “prediction of first initiation of KRT”.	9. Thanks. I have amended this to “Model discrimination for prediction of the sensitivity outcome of non-rebounding eGFR <15 was good (Table 2) but inferior to that observed in the primary analysis predicting first initiation of KRT”.
10. Since there are differences in observed likelihood of kidney failure by sex (as well as diabetes status, age), I would be interested to know if there was any difference in performance of this new equation (and KFRE) in sex subgroups.	10. Thanks, nice idea. I have added further subgroup analyses, for both age (<80, ≥80) and sex, both (i) comparing discrimination performance by subgroups (supplementary table 5), and (ii) assessing calibration by subgroups (supplementary figure 10). Following this, I have added some brief text to Results section on “model performance”, and Discussion section on “strengths and weaknesses of the study”.
11. In my opinion, the manuscript could advocate more strongly for better testing for albuminuria, as this remains an important	11. Thanks, agree. The editor has requested addition of a concluding paragraph at the end of the manuscript so I have added more emphasis on the importance of ACR testing to that paragraph, while stating that this equation provides a welcome alternative to the KFRE in the case of missing ACR data.
	12. Analysis code can be found at: https://github.com/faye-cleary/SCREAM-risk-prediction. I have added this link to the methods-statistical analysis section of the manuscript.
	13. At present, this equation has only be shown to perform well in Sweden, so it would not be suitable to provide an automatic estimation tool for global use at this stage. The equation needs to be validated in other settings before more

marker of risk and response to treatment even beyond its utility in the KFRE.<br><br>12. Do the authors plan to share analysis code?<br><br>13. Have the authors considered creating e.g. a shiny app for their new risk score?	global adoption can be recommended. Risk prediction equations for use in medical practice are regulated as medical devices, requiring regulator approval for distribution and regular oversight and management.

Reviewer 3 (Dr. Andrew John Bentall, Mayo Clinic) comments	Responses
The authors set out to use the Stockholm database for patients without urinary albumin using clinical risk factors for the development of End stage kidney disease as an alternative to KFRE equation. Development population was 85,000 and the validation cohort was 28,000. There were 854 events across both groups. 0.8% in the development cohort and 0.4% in the validation cohort.<br><br>1. Why were the differences in KRT used rather than eGFR <15ml/min which could increase endpoint number? I see in the discussion and figures use of eGFR as an endpoint but do not see the methodology in the paper of this described.<br><br>2. The SCREAM population is almost 50% of the population were over 80 years old and the majority of the population had CKD stage 3a 45 -60ml/min. The authors should determine the proportion of patients in each age group with each different GFR and endpoint.	1. 2 outcomes were studied, which are described in the methods section on outcomes (primary outcome: first initiation of KRT; sensitivity outcome: non-rebounding eGFR <15). Statistical analyses were carried out for both outcomes and are reported for both outcomes.<br>KRT was considered as primary outcome due to the clinical importance to patients and the healthcare system and reliable measurement of such an outcome using registry data. It is also consistent with outcomes used in previous studies (such as KFRE development).<br>A major challenge with an eGFR-based endpoint is that frequency and timing of testing for eGFR impact our ability to identify sustained eGFR < 15 and testing practices may vary substantially between patients in a non-random way, i.e. based on factors which influence risk. (Please see supplementary table 3.) This may lead to both incomplete measurement of the outcome and differential misclassification bias. (For example, predicted risks will potentially appear artificially lower among those with less frequent tests.) In the worst-case scenario, this may lead to an inaccurate risk equation with poor model fairness.<br><br>2. Thanks. It is true that we studied a vast CKD population including patients with eGFR tests carried out across all care settings (excluding inpatients), therefore capturing a relatively high proportion of earlier stage CKD and older age. Our aim was to develop an equation suitable for use in all patients with CKD stages 3-4. Considering your point and comments from other reviewers, I have added additional subgroup

	analyses by age (supplementary table 5, supplementary figure 10) to see if the model breaks down in those aged over 80 and/or those with earlier stage CKD. C statistics showed relatively reduced model performance in those aged >80, which appears to be comparatively worse in those also with earlier stage CKD. (Please note that reductions in the variability of the sample in subgroups are responsible for some of the reduction in discrimination.) Please see the footnote of supplementary table 5 which shows the proportion of patients falling in each age by CKD stage category. Calibration plots also showed over-estimation of risks in those aged >80 (likely reflecting reduced uptake of KRT in such patients). I have added some comments on these new results to Results section on model performance and Discussion section on strengths and weaknesses of the study.
3. Whilst there was discussion on proportional analysis which would help to predict end stage kidney disease, death without dialysis is also an important endpoint.	3. While we recognise that CKD-related death without dialysis is an important outcome for patients, this was not the focus of our work. CKD -related death is also challenging to define. Our primary aim was to provide a new tool to support better prioritisation of care for patients with CKD with the potential to delay CKD progression and reduce the growing burden of ESKD requiring KRT which present such a heavy burden to both patients and healthcare systems. We compare our work to the existing KFRE adopted in routine clinical care which is based on KRT events, not deaths.
4. The authors describe the data of those with higher risk of KTR being younger in Supplemental Table 6.	4. Yes, on average increased age is associated with reduce risk of KRT, which is likely driven by the very elderly not being suited to KRT or declining KRT. This is also demonstrated in the final analysis model, by the hazard ratio for age (Table 2). This inverse association between age and KRT risk among patients with CKD is consistent with the KFRE.
5. Given that this study was submitted in 2025, the eGFR equation should use the CKD EPI 2021 formula, as it will also apply better to other populations if used going forward. There is likely little numeric difference particularly in a Scandinavian cohort, but is more contemporaneous to current practices. No ethnicity is reported in the Table 1 demographics.	5. We could not report ethnicity data in Table 1, as such data are not collected in Sweden by law. For the purposes of this analysis, all patients were assumed Caucasian. We would respectfully prefer to continue using the CKD-EPI 2009 equation in our study. We argue that the CKD-EPI 2021 equation was not developed/used at the time the data was collected. In a previous study using this same data source (PMID: 35689668) we found that transitioning from the 2009 to the 2021 equation in this predominantly white north European population would importantly impact on CKD prevalence, reclassifying many patients to a higher KDIGO eGFR category. In part based on
6. Using historical eGFR linear regression to demonstrate eGFR decline in prior 3 years is hard value to use in clinical practice – requires	

increased data that may not be present in the majority of patients.<br><br>7. in epidemiological studies difficult to determine, but<br>GFR<15 – described in starting KRT – 25% died, but those not starting 68% died. This is likely the result of end of life conversations and thus KRT might not be the best endpoint for sensitivity. Understanding the differences in these populations would help the reader.<br><br>8. The risk models demonstrate that the younger the patient (median age 64-67) are higher risk of reaching KTR stage. Supporting the fact that >80yrs old with CKD stage 3a are not at significant risk of progression.<br><br>9. Supplementary Figure 18 shows that using KFRE is still better than new model at 5% risk per NICE guidelines.	this work, the European Renal Association (ERA) issued an official statement dis-recommending the use of the CKD-EPI 2021 equation in Europe (PMID: 36069913), because of the larger biases imposed in White individuals. This decision follows the 2024 KDIGO guideline recommendation that each country/region should use the eGFR equation that best approximates to mGFR in their patients (PMID: 38490803).<br><br>6. We appreciate that reliance on data depicting historical eGFR decline may limit utility of the equation in healthcare systems with limited data. However, at least 2 eGFR results are generally required in clinical practice to confirm chronicity of decreased eGFR compatible with CKD. The requirement of 3 eGFR results seems reasonable in mature healthcare record systems. In our dataset, the median number of eGFR results up to the index date was 10 and 97.6% of patients in our cohort identified between 2010-2018 had at least 3 historical eGFR results (Table 1). We did not apply a restriction on the timeframe for eliciting these 3 measures in our dataset (besides the fact that at least 2 results must be separated by 90+ days to confirm evidence of CKD). Given the statistical significance of this variable in our analysis, we decided to include this variable in our model to improve prediction of risks of KRT. We do not rule out the suggestion that this model could be compared in the future to an equivalent model without the inclusion of the historical eGFR decline variable if data availability was an issue in some settings, or if differences in frequency of measurement between settings (and resulting estimate of decline) impacted transportability of the model.<br><br>7. I think this comment comes from the following manuscript sentence in results sub-section agreement between outcomes: “Those with CKD5 who went on to start KRT mostly survived (25% died; median 8 days between eGFR<15 and death), while those not starting KRT mostly died (68% died; median 25 days between eGFR<15 and death).”<br>I agree with the comment that among those with CKD stage 5, higher death rates in those not starting KRT are likely due to complex factors relating to uptake of KRT and end of life care. For example, KRT may be rejected on the basis of a coinciding high risk of death (possibly due to older age or risk factors unrelated to CKD such as cancer) or patient preference to avoid KRT which inevitably leads to death. I have added some text to the results section to emphasise this. (Some patients with CKD stage 5 may not have been eligible for KRT, with many patients starting KRT at lower eGFR levels.)

10. Whilst the 3 variable model does not perform as well as the full model, it would be easier to implement in PCP for screening for CKD progression than the full model.0.936 vs. 0.944 is excellent model prediction value.<br><br>11. In summary, the model performs well statistically but the number of variables would be hard to implement in clinical practice. There is not a significant improvement on the validation of the KFRE equation, but a simpler 3 riskf actor equation could improve risk stratification in the population and lead to further testing including urinary albumin where a more accurate KFRE formula could then be applied.<br><br>Questions<br>1. Authors use the UK system as an introduction, but the study is performed in a Swedish cohort with different healthcare systems. Populations may or may not be different given immigration, multi-ethnic UK population and increased CKD in non-Caucasian UK population. Hard to directly extrapolate.<br><br>2. Given that the lead authors are from the UK, could the validation have been done in a UK primary care database? The age of the population in the SCREAM database is a potential weakness.	Regarding the point about outcome sensitivity, we recognise that a potential drawback of the KRT outcome (as opposed to an eGFR-based outcome) is that as it does not capture a purely biological state, being influenced by patient and physician preference. The KRT outcome requires acceptance of KRT rather than conveying absolute clinical need for KRT, potentially leading to measurement inconsistency between patients. We see this as a minor limitation, in consideration of the strengths of the outcome, such as clinical importance. The pros and cons of such measures are discussed in detail elsewhere (DOI: 10.1016/j.kint.2022.09.020).<br><br>8. Agree. Table 2 shows decreased risk of KRT (and eGFR outcome) with increasing age. It is correct that patients aged >80 with CKD stage 3a are among those at lowest risk of KRT.<br><br>9. Yes, we recognise that KFRE has marginally improved discrimination over our new equation. However, KFRE requires data on uACR which is frequently not available, whereas our equation does not.<br><br>10. This is a fair and valuable point, reflecting results from supplementary table 7. To emphasise this, I have added the following text to the new concluding paragraph of the discussion. “In the absence of complete data on predictors included in our final model, the 3-variable KFRE is an alternative which demonstrates good model performance in the population studied.”<br><br>11. This is a reasonable point. However, we believe that the model variables would be relatively easy to harness in most developed healthcare systems using electronic healthcare records. It may also be possible to automate risk estimation using software tools, if the model were to be accepted for adoption in clinical practice. We accept that the 3-variable KFRE (age, sex, eGFR) is likely to be an attractive alternative to our new equation in the absence of complete data on our model predictors and uACR data. We are sceptical that uACR testing would be improved by risk estimation with 3-variable KFRE, given very poor uACR testing in clinical practice currently despite clinical guidelines recommending uACR testing.

1. Thank you for this point. We recognise that further validation in the UK is needed before the equation can be recommended for use in the UK, where indeed there may be differences in

	patient characteristics and healthcare practices between the UK and Sweden. The benefits of using this Swedish data resource were its vast and complete coverage of patients with eGFR tests compatible with CKD stages 3-4 in the healthcare records of patients in Stockholm region, and completeness of the data across multiple care settings. Electronic healthcare records data in the UK are commonly more fragmented (for example eGFR tests may only be available for analysis if ordered in primary care). Unfortunately, we did not have access to a suitable dataset (with linkage to registry data for kidney failure outcomes) in the UK for this work at the time of analysis. We would like to validate the developed equation in the UK in due course, with re-calibration if required.<br>2. We did intend to carry out additional validation in the UK, but data were not readily available to support this at the time of submission. We did not want to delay publication of the equation which is well validated in Sweden. We plan to validate the equation in the UK in the future, but this does not impact it's more immediate potential clinical utility in Sweden, given that it has been developed and validated using the routine healthcare data of all patients with confirmed evidence of CKD stages 3-4 in the entire region of Stockholm. The age of the population reflects patients identified with evidence of CKD stages 3-4 in Stockholm region, and therefore the equation could be used in Swedish patients with the same age range.

Reviewer 4 (Prof. Rafael Perera, University of Oxford) comments	Responses
Stats Review:<br><br>The present manuscript provides results from a study that developed and internally validated a risk prediction model for kidney failure (KRT – Kidney Replacement Therapy) and a sensitivity outcome of 'non-rebounding eGFR<15 or KRT. Crucially, this risk prediction model does not include albumin:creatinine ratio (uACR) as the recording of this biomarker is only available for a minority of patients with CKD. This derivation and validation utilise data from over 116,000 participants in Sweden (the Stockholm Creatinine Measurements (SCREAM) database).<br><br>The manuscript is generally well-written, and the model is likely to be useful in systems with mature healthcare records, as the automatic estimation of these risk scores could be easily employed.	

There are some queries and clarifications about the approach taken and the reporting of the study that would benefit from addressing:<br><br>1. A significant proportion of participants died during the study. This is not the primary outcome or part of a composite one. Given this, it is unclear what the impact of this 'competing risk' is on the estimated model. It is unclear whether this would likely create bias or impact the calibration of the risk score. Further analyses taking death into consideration might be required.	1. The modelling approach we employed which censors for deaths is the same approach taken as in development of the KFRE. Estimated risks in the next 5 years are based on an assumption that death will not occur during this time (i.e. "what would be the risk of kidney failure in the next 5 years if the patient does not die during this time?"), and that the risk of death is independent of the risk of kidney failure. I have added some words to the methods-statistical analysis section to clarify this. There are pros and cons to this approach, as compared to a model which accounts for the competing risk of death. A benefit of the approach is that by not attenuating estimated risks of kidney failure to account for a high competing risk of death, patients will not be "penalised" due to a high risk of death in terms of opportunity for being progressed for further care, when this depends on the risk estimate. Likewise, the risk estimate is expected to be reasonable among those who do not die, of which there will be many, and who deserve to access further care. A potential drawback is interpretability to patients and clinicians, where the absolute estimated risk of kidney failure may not be a true reflection of a patient's likelihood to ever receive KRT if they have a competing high risk of death. The interpretation of the estimated risk (expected risk in next 5 years, assuming the patient does not die) may also be less suited to scenarios such as practical planning for dialysis initiation. As stated in the discussion, in international cohort analyses, on average there was not much difference in predicted risks between models for kidney failure that take account of death and those that do not, although we recognise that the bias is likely to be worse in patients with high risk of death. In this study, our focus was to provide a new model capable of improving decision-making on prioritisation of care (e.g. referral to specialist care), with specific comparison with the KFRE (which also does not adjust for competing risk of death) and we do not intend to carry out further analyses in a competing risks framework. However, we recognise the potential value of such an approach in future work and other studies.
2. Variable selection using 'backward selection' has generally been superseded by regularisation techniques (e.g. Lasso) . Please provide a rationale/justification for backward selection.	2. The goal of our analysis was to arrive at a simple and clinically informed model to allow risk estimation in all patients with CKD stages 3-4. Our dataset was large, representative, comprehensive, and was not affected by overfitting (observed through near-negligible observed optimism of modelling strategy in internal validation bootstrapping procedures). We also selected candidate predictors based on clinical reasoning, ensuring that any variables included in the model had a strong clinical basis for inclusion in the final model, further reducing the risk of inclusion of spurious or clinically unimportant predictors

	(overfitting). By starting from the full model including all candidate predictors, and removing variables one-by-one that contributed little to model performance, there was also a low risk of failing to include important predictors (underfitting). An added benefit of backward selection (compared to LASSO) is clinical interpretability of reported hazard ratios, reflecting observed adjusted associations occurring in the data. Furthermore, we believe that the precise model building strategy used is less important than the validity, performance, and potential clinical utility of the final model achieved. I have added a few brief comments to the methods section justifying the rationale for backward selection.
3. There was a lack of clarity about the selection of the period under study. There is a table that aims to clarify this, but a brief explanation of the rationale is still required (apologies if I have missed this).	3. Different possible cohort entry periods were considered, comparing number of events (for power), availability of predictor data such as >= 3 x eGFR test results (data completeness), availability of uACR (to enable comparisons with KFRE) and calendar period (indicating the likelihood of data to reflect current care practices). We selected the most recent cohort with high completeness for historical eGFR test results and sufficient power to produce an accurately estimated model. I have added some text to methods-participants section to clarify this.
4. The event counts per variable appear to be relatively large and likely sufficient; however, it would be useful to understand whether a formal sample size estimation was used and the parameters associated with it.	4. As stated in the methods sample size section, the study was based on feasibility and no formal sample size calculations were carried out. There were 52 events per candidate predictor variable, which exceeds rough rules of thumb of approximately 10 events per variable.
5. A critical analysis comparing those with uACR measured to those without suggests some relevant differences in risk levels. Some of this is managed by the relevant other variables included in the model (beyond those included in KFRE). The authors suggest this might be related to medication practices being used as proxies for uACR. Worth discussing how transportable this specific aspect is to other settings outside Sweden.	5. This is a very fair and important point, and if prescribing practices differ between Sweden and other settings, then this could lead to issues of transportability. I think we have already addressed this adequately within the manuscript. We mention the issue in discussion section “strengths and weaknesses in relation to other studies, discussing important differences in results”. We state “our equation has not been validated in other CKD cohorts in different geographical locations, limiting widespread use. Where KFRE includes variables which are likely to be measured fairly consistently (age, sex, eGFR, uACR), our equation includes variables which may be subject to measurement variation in different settings and some which depend on care practices which may change over time. This may impact transportability and longevity of our equation.” We later state “External validation will be required in any other settings of intended use (assessing transportability).”
6. Please expand on how the bootstrap resampling was used as part of the first internal validation using the development cohort. Were these used to determine a better overall estimate of the models or for model selection? Please specify. Related to this point, please justify the use of 200 bootstrap samples.	6. We used bootstrap resampling to compute the optimism-corrected Harrell’s C statistic in the development dataset, reflecting guidance provided

7. When comparing linear predictors, consider using alternative methods for testing agreement, such as Bland-Altman plots. These could help identify systemic biases within the range of predictions.<br><br>8. For Supplementary Figure 6, the event rates between the development and validation cohorts differ significantly. Could this be explored in the Discussion?	in the TRIPOD explanation and elaboration document (https://doi.org/10.7326/M14-0698). Due to our large dataset size, average optimism (averaged differences between C statistic in full sample and bootstrap samples) was very close to zero showing a robust modelling strategy which was not over-optimistic (not susceptible to overfitting), and a larger number of samples was not required to improve accuracy of estimation any further. I have added these clarifications to the methods statistical analysis section.<br><br>7. Ok, thanks, good idea. I have added a Bland-Altman plot. (It did not make sense to plot/compare the linear predictions by themselves, since the baseline hazard estimates differ between our equation and the KFRE, impacting risk estimation. (The KFRE is mis-calibrated in our study population.) Therefore, we plot for a logit-transformation of the predicted risks [approximately normally distributed] (see new Supplementary Figure 15) and provide an additional scatter plot of predicted risks compared between the 2 models (Supplementary Figure 16:A). I have added a few brief comments on the results to Results section “Comparison with 4-variable KFRE”. Essentially, the figures demonstrate/emphasise the consistent under-estimation of KFRE across the risk spectrum (due to miscalibration in the study population).<br><br>8. The reason for the difference in event rates is that the development cohort included comparatively more cases of prevalent CKD and the validation cohort included more cases of incident CKD, by the nature of how data were accrued for individuals over time. Those with longer-standing CKD are more likely to have further progressed disease and higher risk of adverse outcomes than those identified for the first time. The Results section on “outcomes” states: “Event rates were higher in 2010 than subsequent years, due to identifying disproportionately more prevalent CKD cases (vs incident cases) in the first year of follow-up (Supplementary Figures 5-7).” I have added a clause to this sentence to help explain the impact on the event rates in the development and validation cohorts as follows “leading to higher outcome event rates in the development cohort than the validation cohort”. It is important to note that this does not negatively impact model evaluation. In fact, it is a strength of the model that it continues to perform well in a lower risk validation cohort.

Editor (Dr Sophie Cook) Comments to Author	
1. Implications section: They talk about how this could be adopted in Sweden "immediately" and how this is likely to improve healthcare equity	1. I have removed the word “immediately” when stating that the equation could be used in clinical

but don't we need more validation before we can make these claims? The cohort is quite restricted and one of the reviewers is concerned about the age of the participants here.<br><br>2. Please review the end of the manuscript to add a concluding paragraph.<br><br>3. PPI statement needs to just say no patients were involved in the study but they do have patient involvement from a dissemination point of view.	care in Sweden, adding the caveat about this requiring adoption for use in clinical practice.<br>A huge strength of this work is that the dataset used includes all eGFR tests carried out in the Stockholm region of Sweden. This means the developed equation is highly likely to be generalisable to this entire setting, and most likely to the rest of the country. The very large size of the dataset reduced the risk of overfitting, and this was demonstrated by negligible difference in naïve C statistic (apparent performance, 0.941) and optimism-adjusted C statistic (0.941) computed as part of internal validation with bootstrapping procedure. There was also negligible difference between C statistics in the development and validation cohorts (0.941 and 0.944, respectively), despite differences in CKD severity and calendar periods between these 2 cohorts. The high discrimination observed suggests we have developed a powerful tool to rank patients according to risks. Model calibration also remained strong in the validation cohort, within the population and setting studied. It is a misconception that entirely different datasets (development and validation cohorts) are needed to support recommending implementation of a prediction model, provided that the data is representative of future patients to be assessed using the model and that the sample size for model development was large enough to prevent overfitting. Given the widespread coverage of CKD patients in Stockholm in our dataset, it is our opinion that the prediction model developed is ready for use in Sweden, and in particular in the region of Stockholm. We still recommend the model to be validated every few years on more recent data to ensure predictions are still valid, in particular in case care practices change and impact model performance, with re-calibration if required.<br>The cohort is only restrictive in that it is limited to Swedish patients accessing healthcare in the region of Stockholm. In other aspects, the cohort is vast, covering a substantial cross-section of cases of CKD stages 3-4 with different level of severity, and that is not limited to patients with uACR test results (addressing a problem with the existing KFRE). External validation is however required before the equation can be used in any other setting (assessing transportability). I have added further subgroup analyses to address concerns from reviewers about any impact of age on risk prediction.<br><br>2. Thank you. I have added a concluding paragraph at the end of the discussion.<br><br>3. I have amended the PPI statement as suggested.

VERSION 2 – REVIEW

**REVIEWER NAME**	Lees, Jennifer
**REVIEWER AFFILIATION**	University of Glasgow, Institute of Cardiovascular and Medical Sciences
**REVIEWER CONFLICT OF INTEREST**	None
**AI DISCLOSURE**	No
**DATE REVIEW RETURNED**	04-Dec-2025

**GENERAL COMMENTS**	Thanks to the authors for addressing my prior comments. I particularly appreciate the addition of sex (and age) subgroups.<br><br>I have no additional suggestions.

**REVIEWER NAME**	Bentall, Andrew John
**REVIEWER AFFILIATION**	Mayo Clinic, Division of Nephrology
**REVIEWER CONFLICT OF INTEREST**	None
**AI DISCLOSURE**	No
**DATE REVIEW RETURNED**	08-Dec-2025

**GENERAL COMMENTS**	The authors adequately answered my prior reviewer comments and clarified the data points, age risk and inserted into the manuscript descriptions to improve clarity.

**REVIEWER NAME**	Perera, Rafael
**REVIEWER AFFILIATION**	University of Oxford, Primary Care Health Sciences
**REVIEWER CONFLICT OF INTEREST**	None
**AI DISCLOSURE**	No
**DATE REVIEW RETURNED**	11-Dec-2025

**GENERAL COMMENTS**	Many thanks to the authors for answering my queries. These have been addressed fully, and I believe the current version of the manuscript is suitable for publication.<br><br>I have one minor query on their Figure 2 (Calibration plot). The values provided for the quantiles and those appearing in the plot do not appear to match. Please can these be checked? This is also an issue in the calibration plots in the supplementary material. I might be reading these wrong.
